# Supplementary material for: Synergistic effects of FGFR1 and PLK1 inhibitors target a metabolic liability in KRAS‐mutant cancer
Source: EMBO Mol Med. 2021 Aug 8;13(9):e13193. doi: 10.15252/emmm.202013193 (PMC8422071; doi:10.15252/emmm.202013193)
Supplement: Supplementary file 2 — Appendix [file EMMM-13-e13193-s005.pdf]

Appendix Figures and Tables

Synergistic effects of FGFR1 and PLK1 inhibitors target a metabolic liability in *KRAS*-mutant cancer

Zhang Yang[1,2,7], Shun-Qing Liang[1,7], Maria Saliakoura[3], Haitang Yang[1], Eric Vassella[4], Georgia Konstantinidou[3], Mario Tschan[4], Balazs Hegedüs[5], Liang Zhao[1], Yanyun Gao[1], Duo Xu[1], Haibin Deng[1], Thomas M. Marti[1], Gregor J. Kocher[1], Wenxiang Wang[6], Ralph A. Schmid[1]*, Ren-Wang Peng[1]*

Table of content:

Appendix Figure S1

Appendix Figure S2

Appendix Figure S3

Appendix Figure S4

Appendix Figure S5

Appendix Table S1

Appendix Table S2

Appendix Table S3

Appendix Table S4

Appendix Table S5

Figure S1

A

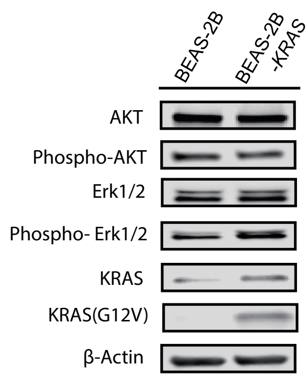

B

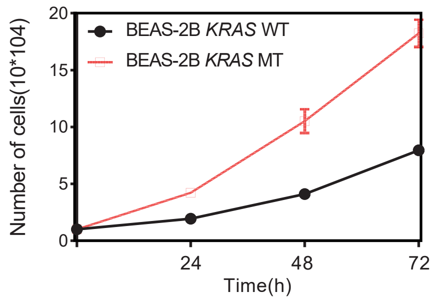

C

BEAS-2B-KRAS

Drug paired with BI2536	Target	Fa	CI
AZD4547	FGFR1-3	0.97	0.25
Ponatinib	RTKs	0.74	0.38
QNZ(EVP4593)	NF-κB	0.69	0.31
Fasudil	ROCK	0.88	0.57
Onalosptib	HSP-90	0.76	0.63
R428	AXL	0.48	1.01
Afatinib	EGFR	0.55	0.97
AZD5363	AKT1-3	0.53	0.93
NVP-BEZ235	PI3K/mTOR	0.45	1.2
Rapamycin	mTOR	0.52	0.98
Trametinb	MEK1/2	0.47	1.01
Sorafenib	RAF	0.56	0.9
Palbociclib	CDK4/6	0.55	0.91
ABT-737	Bcl-2/Bcl-xL	0.53	0.89
MG132	proteasome	0.6	0.83
SNS-314	AuroraA/B/C	0.51	0.95
Hydroxychloroquine(HCQ)	TLR9/Autophagy	0.44	1.09
MK-1775	WEE1	0.53	0.95
GDC-0575	Chk1	0.5	0.99
RGF-966	HDAC3	0.49	1.01
Olapanib	PARP1/2	0.59	0.83

BEAS-2B

Drug paired with BI2536	Target	Fa	CI
AZD4547	FGFR1-3	0.51	1.12
Ponatinib	RTKs	0.57	0.81
QNZ(EVP4593)	NF-κB	0.63	0.78
Fasudil	ROCK	0.56	0.93
Onalosptib	HSP-90	0.66	0.65

D

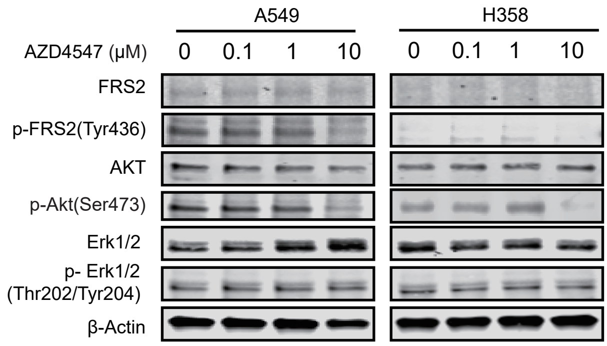

E

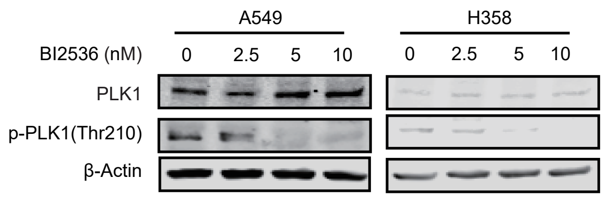

F

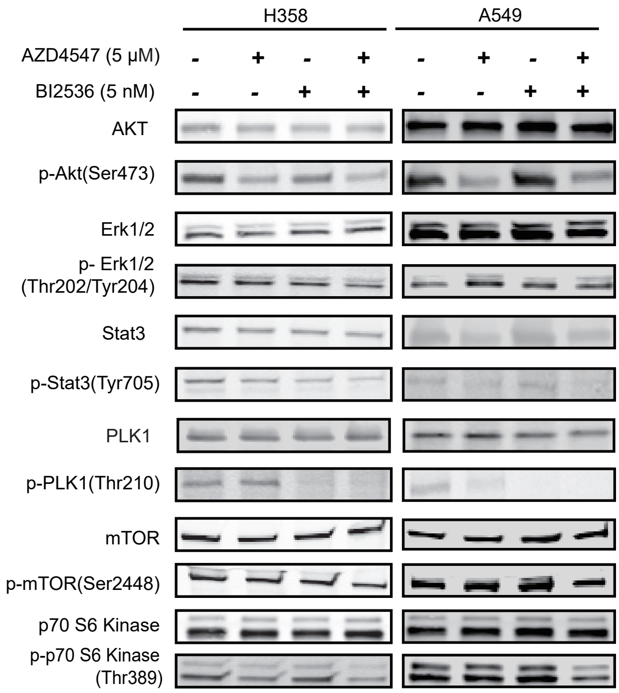

Appendix Figure S1. Chemical screens identify synthetic lethal drugs with PLK1 inhibitor BI2536 in mutant *KRAS*-expressing cells

A, B, Immunoblots (A) and growth curve (B) of BEAS-2B and BEAS-2B-KRAS cells. (B) Data were from three independent experiments (n=3), error bar: SD.

C, The Fa and CI values of individual drug combinations (compounds plus BI2536 used at their IC_{50}) in BEAS-2B-KRAS cells. Shown underneath are the FA and CI values of the candidate compounds in combination with BI2536 in BEAS-2B cells. The results are average of two independent experiments (n=2).

D, Immunoblots of A549 and H358 cells treated for 2 hours with AZD4547 of the indicated concentrations.

E, Immunoblots of A549 and H358 cells treated for 2 hours with BI2536 of the indicated concentrations.

F, Immunoblots of H358 and A549 cells treated with vehicle (DMSO), AZD 4547(5 µM) and BI2536 (5 nM), alone or in combination for 24 hours.

Figure S2

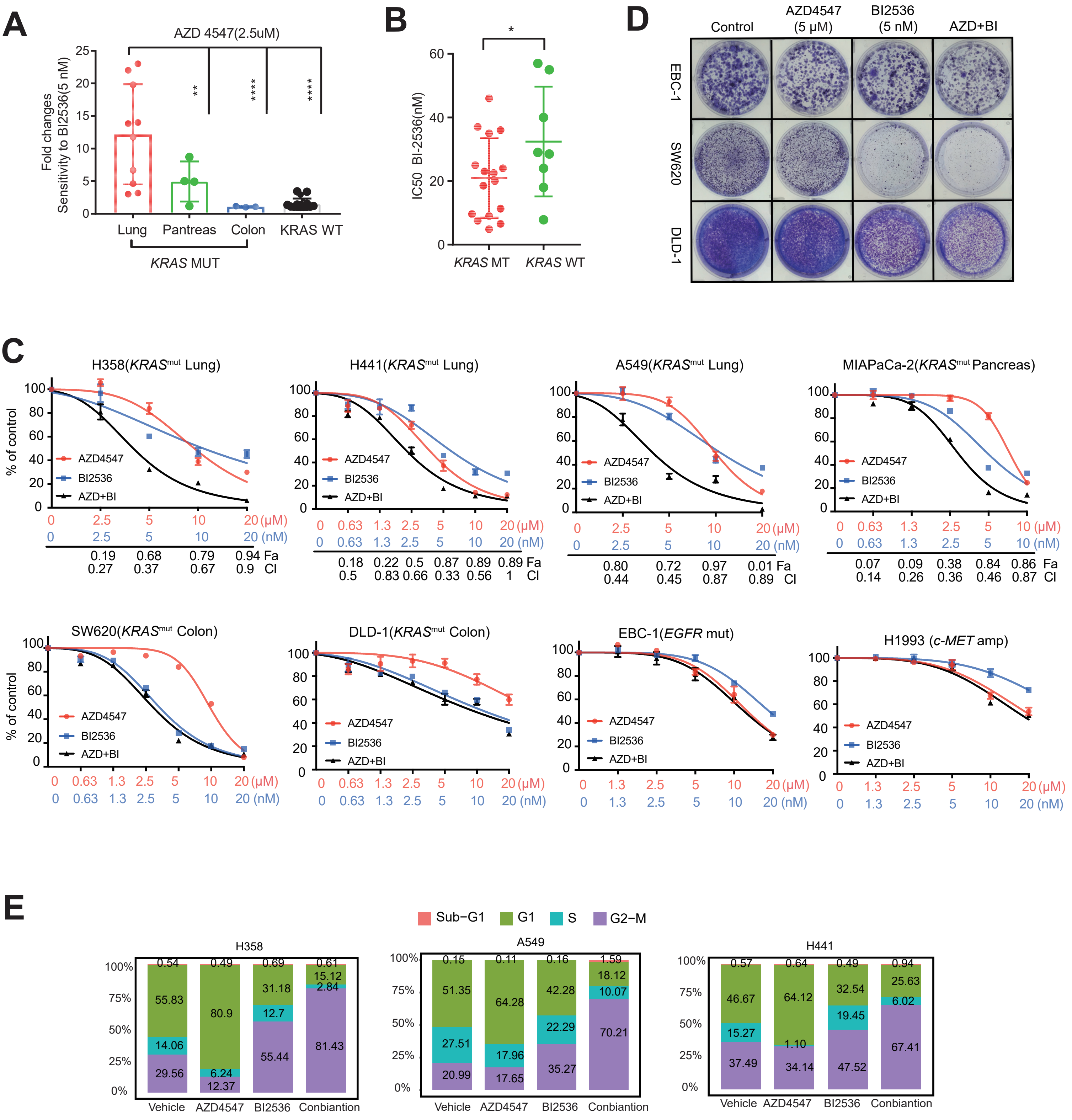
A
AZD 4547(2.5uM)
Fold changes
Sensitivity to BI2536(5 nM)
25
20
15
10
5
0
**
****
****
Lung
Pantreas
Colon
KRAS WT
KRAS MUT
B
*
IC50 BI-2536(nM)
60
40
20
0
KRAS MT
KRAS WT
D
Control
AZD4547 (5 μM)
BI2536 (5 nM)
AZD+BI
EBC-1
SW620
DLD-1
C
H358(KRASmut Lung)
H441(KRASmut Lung)
A549(KRASmut Lung)
MIAPaCa-2(KRASmut Pancreas)
% of control
AZD4547
BI2536
AZD+BI
0 2.5 5 10 20 (μM)
0 2.5 5 10 20 (nM)
0.19 0.68 0.79 0.94 Fa
0.27 0.37 0.67 0.9 CI
0 0.63 1.3 2.5 5 10 20 (μM)
0 0.63 1.3 2.5 5 10 20 (nM)
0.18 0.22 0.5 0.87 0.89 0.89 Fa
0.5 0.83 0.66 0.33 0.56 1 CI
0 2.5 5 10 20 (μM)
0 2.5 5 10 20 (nM)
0.80 0.72 0.97 0.01 Fa
0.44 0.45 0.87 0.89 CI
0 0.63 1.3 2.5 5 10 (μM)
0 0.63 1.3 2.5 5 10 (nM)
0.07 0.09 0.38 0.84 0.86 Fa
0.14 0.26 0.36 0.46 0.87 CI
SW620(KRASmut Colon)
DLD-1(KRASmut Colon)
EBC-1(EGFR mut)
H1993 (c-MET amp)
0 0.63 1.3 2.5 5 10 20 (μM)
0 0.63 1.3 2.5 5 10 20 (nM)
0 1.3 2.5 5 10 20 (μM)
0 1.3 2.5 5 10 20 (nM)
E
Sub−G1
G1
S
G2−M
H358
100%
75%
50%
25%
0%
0.54 0.49 0.69 0.61
55.83 80.9 31.18 15.12
14.06 6.24 12.7 2.84
29.56 12.37 55.44 81.43
Vehicle AZD4547 BI2536 Conbiantion
A549
0.15 0.11 0.16 1.59
51.35 64.28 42.28 18.12
27.51 17.96 22.29 10.07
20.99 17.65 35.27 70.21
Vehicle AZD4547 BI2536 Conbiantion
H441
0.57 0.64 0.49 0.94
46.67 64.12 32.54 25.63
15.27 1.10 19.45 6.02
37.49 34.14 47.52 67.41
Vehicle AZD4547 BI2536 Conbiantion

Figure S2

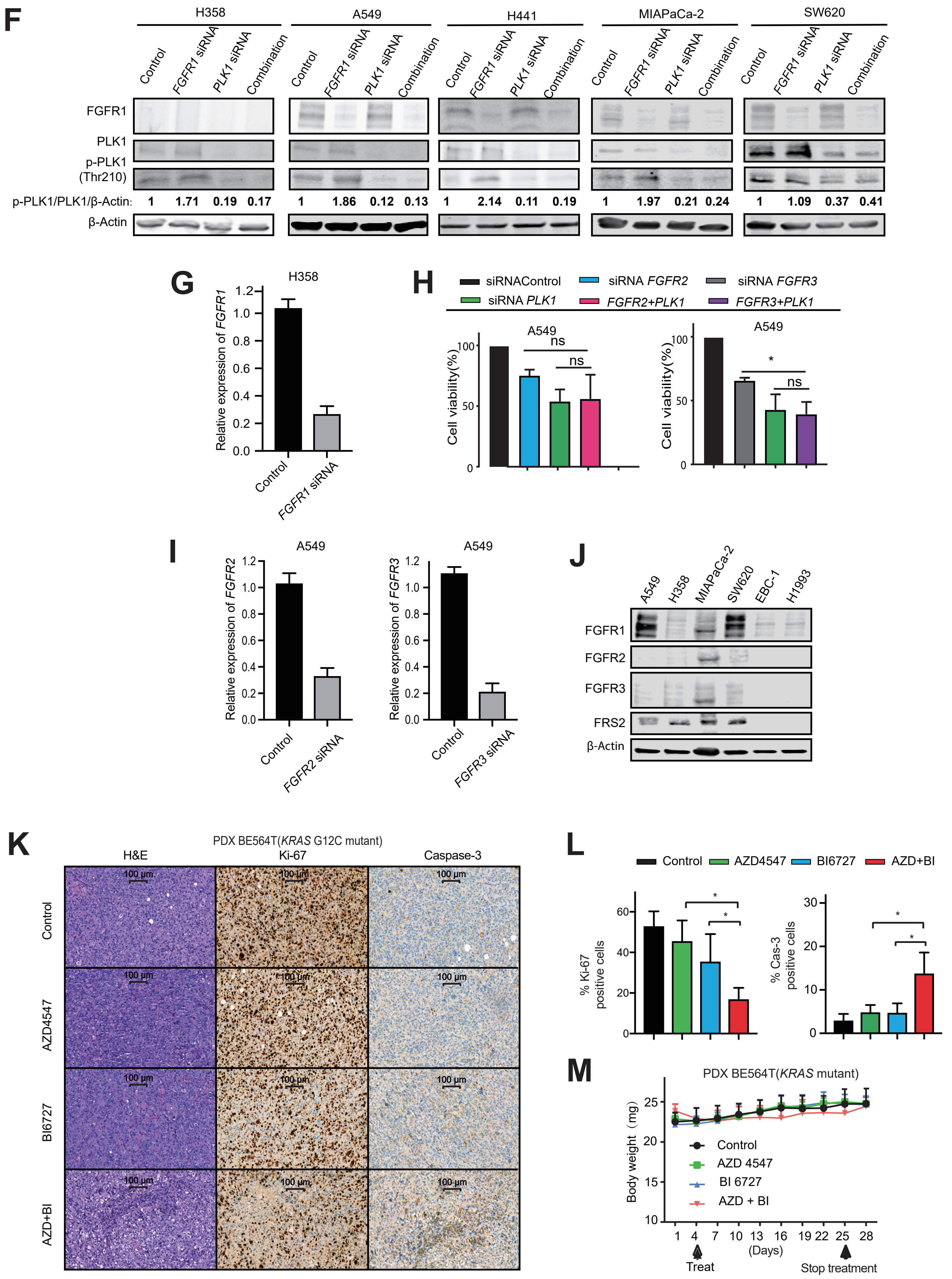
F
H358
A549
H441
MIAPaCa-2
SW620
Control
FGFR1 siRNA
PLK1 siRNA
Combination
FGFR1
PLK1
p-PLK1
(Thr210)
p-PLK1/PLK1/β-Actin:
1 1.71 0.19 0.17
1 1.86 0.12 0.13
1 2.14 0.11 0.19
1 1.97 0.21 0.24
1 1.09 0.37 0.41
β-Actin
G
H358
Relative expression of FGFR1
1.2
1.0
0.8
0.6
0.4
0.2
0.0
Control
FGFR1 siRNA
H
siRNAControl
siRNA FGFR2
siRNA FGFR3
siRNA PLK1
FGFR2+PLK1
FGFR3+PLK1
A549
Cell viability(%)
100
50
0
ns
ns
*
I
A549
Relative expression of FGFR2
Relative expression of FGFR3
Control
FGFR2 siRNA
FGFR3 siRNA
J
A549
H358
MIAPaCa-2
SW620
EBC-1
H1993
FGFR1
FGFR2
FGFR3
FRS2
β-Actin
K
PDX BE564T(KRAS G12C mutant)
H&E
Ki-67
Caspase-3
Control
AZD4547
BI6727
AZD+BI
100 μm
L
Control
AZD4547
BI6727
AZD+BI
% Ki-67 positive cells
60
40
20
0
% Cas-3 positive cells
30
20
10
0
*
*
M
PDX BE564T(KRAS mutant)
Body weight (mg)
25
20
15
10
Control
AZD 4547
BI 6727
AZD + BI
1 4 7 10 13 16 19 22 25 28
(Days)
Treat
Stop treatment

Appendix Figure S2. Combined inhibition of FGFR1/PLK1 synergizes in *KRAS*-mutant lung and pancreatic cancer cells

A, KRAS-mutant and wild-type cancer cells treated with AZD4547 (2.5 µM) alone or combined with BI2536 (5 nM). The fold change in sensitivity (percentage of proliferation inhibition) to drug combination versus single treatment was shown. Data presented are average of two independent experiments (n=2); error bar: SD. ***P*<0.01, *****P*<0.0001 by unpaired two-tailed t-test.

B, The IC50 value of BI2536 (72 h treatment) in *KRAS*-mutant and wild-type lung, pancreatic and colon cancer cell lines (n=24). **P*<0.05 by unpaired two-tailed t-test. ; error bar: SD.

C, Dose-response curves of *KRAS*-mutant cancer cells (H358, H441, A549, MIAPaCa-2 and SW620) and *KRAS* wild-type NSCLC cells (EBC-1 and H1993) to AZD4547 (FGFR inhibitor) and BI2536 (PLK1 inhibitor), alone or combination. Data are presented as mean ± SD. (n=3).

D, Clongenic assay of *KRAS*-mutant colon cancer cells (SW-620 and DLD-1) and *KRAS*-wild-type lung cancer cells (EBC-1) treated with control (DMSO), AZD4547 (5 µM) and BI2536 (5 nM), alone or combination.

E, Cell cycle analysis of H358, H441 and A549 cells treated with vehicle (DMSO), AZD4547 (5 µM) and BI2536 (5 nM), alone or combination for 24h. Data were shown as average from three indedependent experiments (n=3).

F, Immunoblots of *KRAS*-mutant lung cancer cells (A549, H358, H441), pancreatic cancer cells (MIAPaCa-2) and colon cancer cells (SW620) transfected with control siRNAs, *FGFR1*- and *PLK1*-specific siRNAs, alone or in combination for 72 h.

G, Quantitative RT-PCR analysis of H358 cells transfected with *FGFR1*-siRNAs or control siRNAs. Relative *FGFR1* expression was shown, with data presented as mean ± SD. (n=3).

H, Cell viability assay of A549 cells transfected with *FGFR2*-、*FGFR3*- and *PLK1*-specific siRNAs or control siRNAs. Live cells were counted by a hemocytometer. Data are presented as mean ± SD. (n=3). *P<0.05 and P>0.05 (ns) by two-way ANOVA with Tukey's multiple comparisons test.

I, A549 cells transfected with *FGFR2-, FGFR3-* or control siRNAs. Quantitative RT-PCR was used to determine *FGFR2* and *FGFR3* expression. Data are presented as mean ± SD. (n=3).

J, Immunoblots of *KRAS*-mutant cancer cells (A549, H358, MIAPaCa-2, SW620, EBC-1 and H1993).

K, L, H&E and IHC analysis for Ki67 and Caspase-3 (K) of a patient-derived xenograft (BE 564T) after the indicated treatment. Quantification of Ki-67- and Caspase-3-positive cells were shown in (L). Original overall magnification, ×400. *P<0.05 by two-way ANOVA with Tukey's multiple comparisons test. Data are shown as mean ± SD (error bar) of three independent experiments (n=3).

M, Body weights of mice bearing PDX tumors (BE 564T) during the treatment. Data are mean of body weights of each group (5 mice/group); error bar: SD.

Figure S3

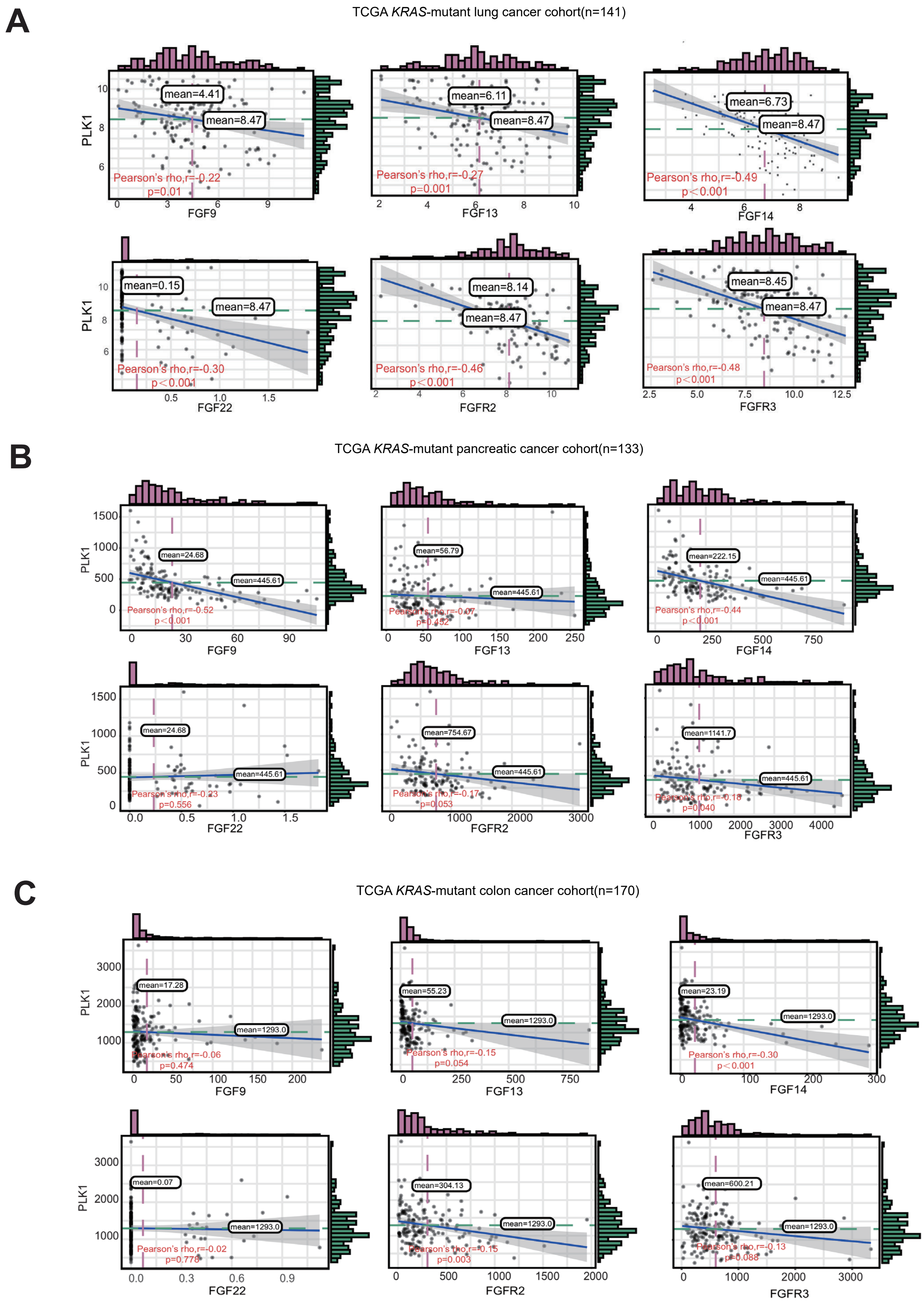
A
TCGA KRAS-mutant lung cancer cohort(n=141)
PLK1
FGF9
mean=4.41
mean=8.47
Pearson's rho,r=-0.22
p=0.01
FGF13
mean=6.11
mean=8.47
Pearson's rho,r=-0.27
p=0.001
FGF14
mean=6.73
mean=8.47
Pearson's rho,r=-0.49
p<0.001
FGF22
mean=0.15
mean=8.47
Pearson's rho,r=-0.30
p<0.001
FGFR2
mean=8.14
mean=8.47
Pearson's rho,r=-0.46
p<0.001
FGFR3
mean=8.45
mean=8.47
Pearson's rho,r=-0.48
p<0.001
B
TCGA KRAS-mutant pancreatic cancer cohort(n=133)
FGF9
mean=24.68
mean=445.61
Pearson's rho,r=-0.52
p<0.001
FGF13
mean=56.79
mean=445.61
Pearson's rho,r=-0.07
p=0.452
FGF14
mean=222.15
mean=445.61
Pearson's rho,r=-0.44
p<0.001
FGF22
mean=24.68
mean=445.61
Pearson's rho,r=-0.23
p=0.556
FGFR2
mean=754.67
mean=445.61
Pearson's rho,r=-0.17
p=0.053
FGFR3
mean=1141.7
mean=445.61
Pearson's rho,r=-0.18
p=0.040
C
TCGA KRAS-mutant colon cancer cohort(n=170)
FGF9
mean=17.28
mean=1293.0
Pearson's rho,r=-0.06
p=0.474
FGF13
mean=55.23
mean=1293.0
Pearson's rho,r=-0.15
p=0.054
FGF14
mean=23.19
mean=1293.0
Pearson's rho,r=-0.30
p<0.001
FGF22
mean=0.07
mean=1293.0
Pearson's rho,r=-0.02
p=0.778
FGFR2
mean=304.13
mean=1293.0
Pearson's rho,r=-0.15
p=0.003
FGFR3
mean=600.21
mean=1293.0
Pearson's rho,r=-0.13
p=0.088

Figure S3

D

TCGA *EGFR*-mutant lung cancer cohort(n=120)

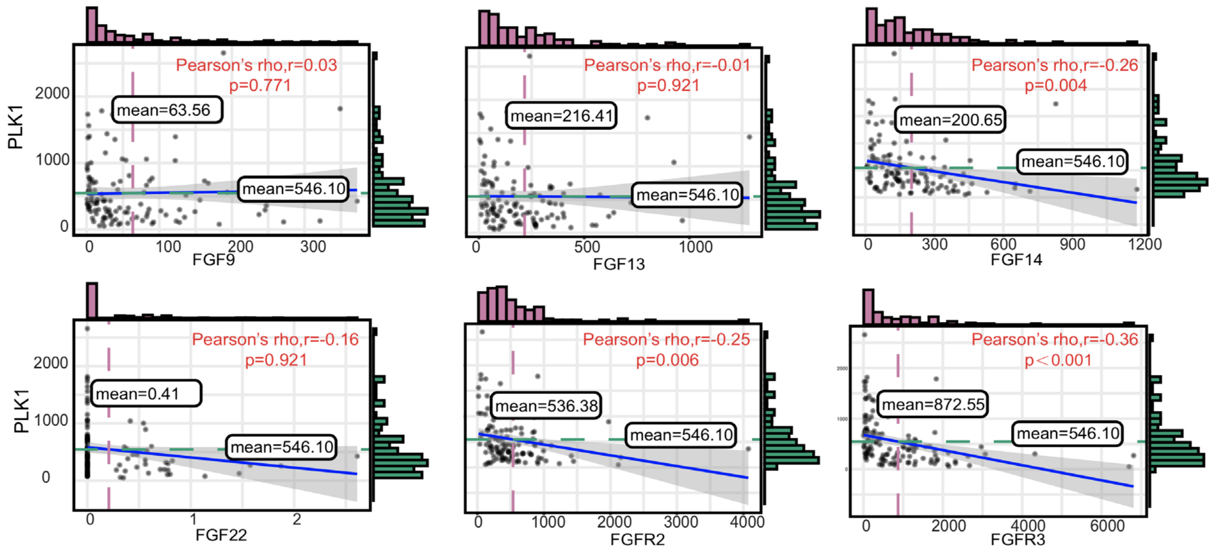

E

TCGA *BRAF*-mutant lung cancer cohort(n=75)

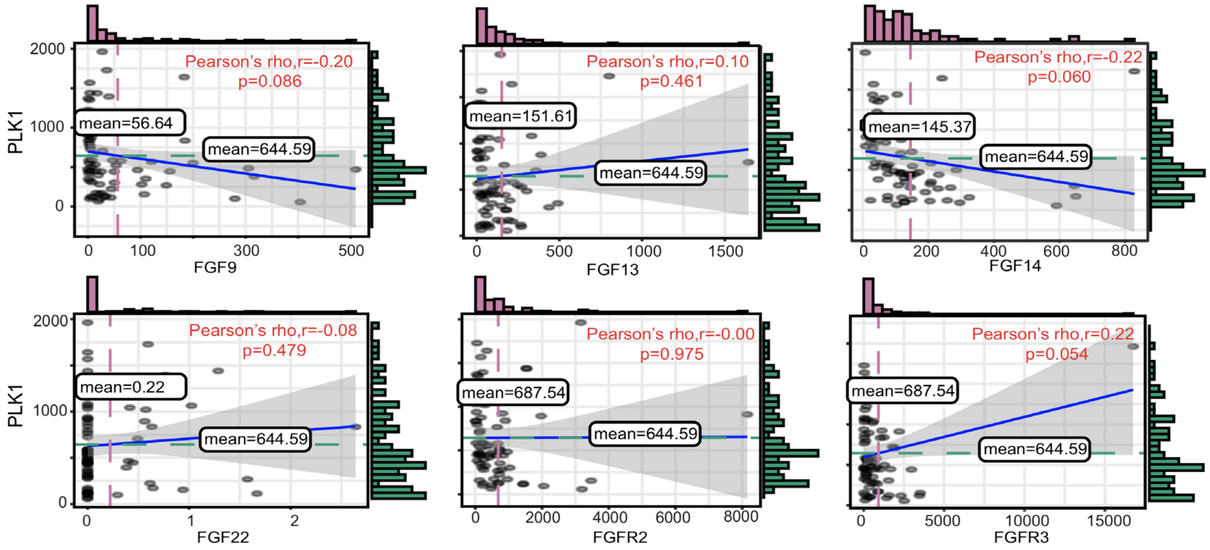

Appendix Figure S3. FGFR1 and PLK1 reciprocate in *KRAS*-mutant cancers

A-E, Correlation analysis between the mRNA level of *PLK1* and that of *FGFR2/FGFR3/FGF9/ FGF13/FGF14/FGF22*. Transcriptomic data of cohorts of patients with (A) *KRAS*-mutant lung cancers (n=141), (B) *KRAS*-mutant pancreatic cancers (n=133), (C) *KRAS*-mutant colon cancers (n=170), (D) *EGFR*-mutant lung cancers (n=120) and (E) *BRAF*-mutant pancreatic cancers (n=75) were downloaded from TCGA. Pearson coefficient and significance (p-value) were determined using R software (Cor. test function).

Figure S4

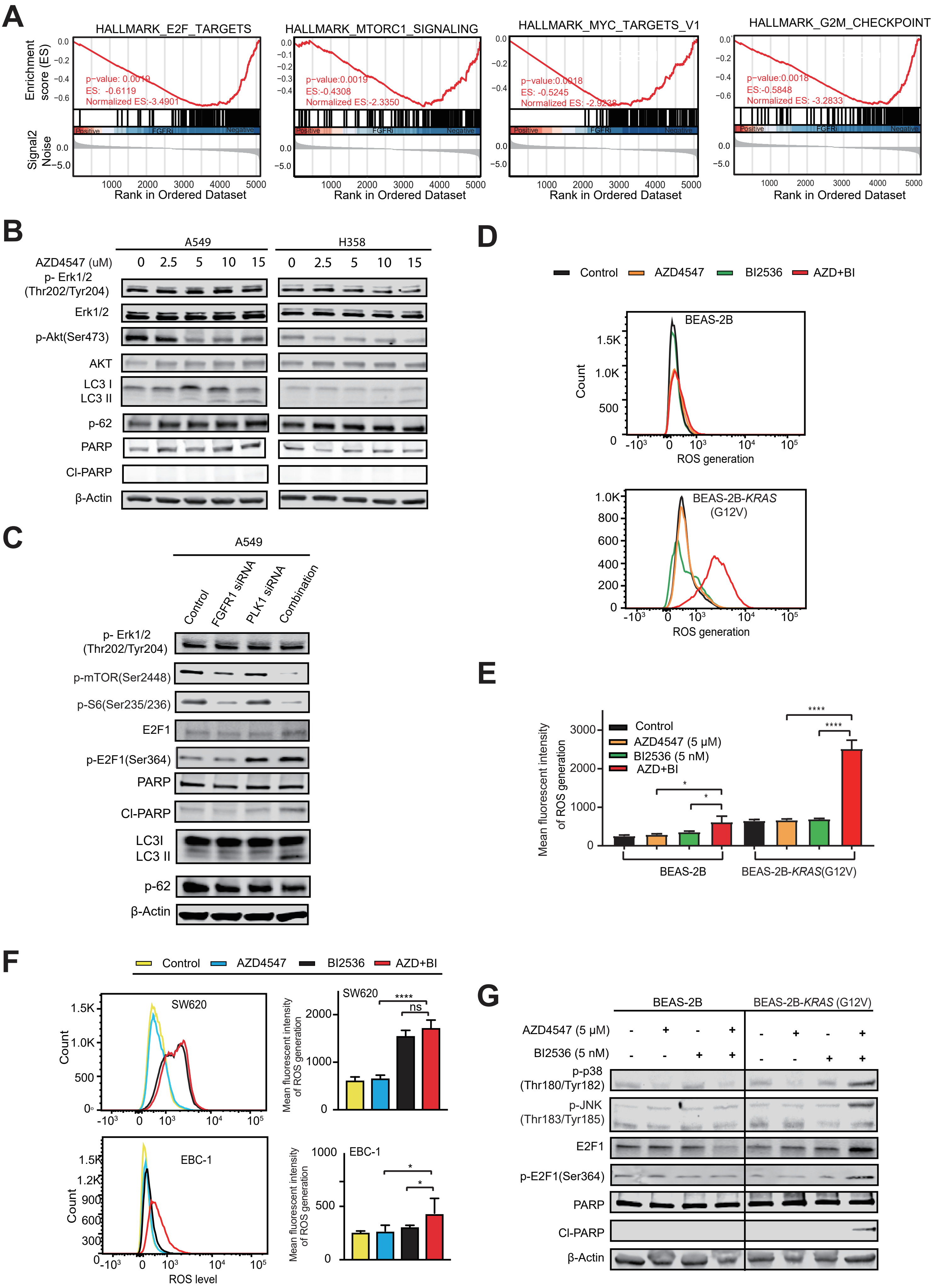
A
HALLMARK_E2F_TARGETS
p-value: 0.0019
ES: -0.6119
Normalized ES:-3.4901
HALLMARK_MTORC1_SIGNALING
p-value:0.0019
ES:-0.4308
Normalized ES:-2.3350
HALLMARK_MYC_TARGETS_V1
p-value:0.0018
ES: -0.5245
Normalized ES: -2.9238
HALLMARK_G2M_CHECKPOINT
p-value:0.0018
ES:-0.5848
Normalized ES: -3.2833
Enrichment score (ES)
Signal2 Noise
Positive
FGFRi
Negative
Rank in Ordered Dataset
B
A549
H358
AZD4547 (uM) 0 2.5 5 10 15 0 2.5 5 10 15
p- Erk1/2 (Thr202/Tyr204)
Erk1/2
p-Akt(Ser473)
AKT
LC3 I
LC3 II
p-62
PARP
Cl-PARP
β-Actin
C
A549
Control
FGFR1 siRNA
PLK1 siRNA
Combination
p- Erk1/2 (Thr202/Tyr204)
p-mTOR(Ser2448)
p-S6(Ser235/236)
E2F1
p-E2F1(Ser364)
PARP
Cl-PARP
LC3I
LC3 II
p-62
β-Actin
D
Control
AZD4547
BI2536
AZD+BI
BEAS-2B
BEAS-2B-KRAS (G12V)
Count
ROS generation
E
Control
AZD4547 (5 μM)
BI2536 (5 nM)
AZD+BI
Mean fluorescent intensity of ROS generation
BEAS-2B
BEAS-2B-KRAS(G12V)
F
Control
AZD4547
BI2536
AZD+BI
SW620
EBC-1
Count
ROS level
Mean fluorescent intensity of ROS generation
ns
G
BEAS-2B
BEAS-2B-KRAS (G12V)
AZD4547 (5 μM) - + - + - + - +
BI2536 (5 nM) - - + + - - + +
p-p38 (Thr180/Tyr182)
p-JNK (Thr183/Tyr185)
E2F1
p-E2F1(Ser364)
PARP
Cl-PARP
β-Actin

Figure S4

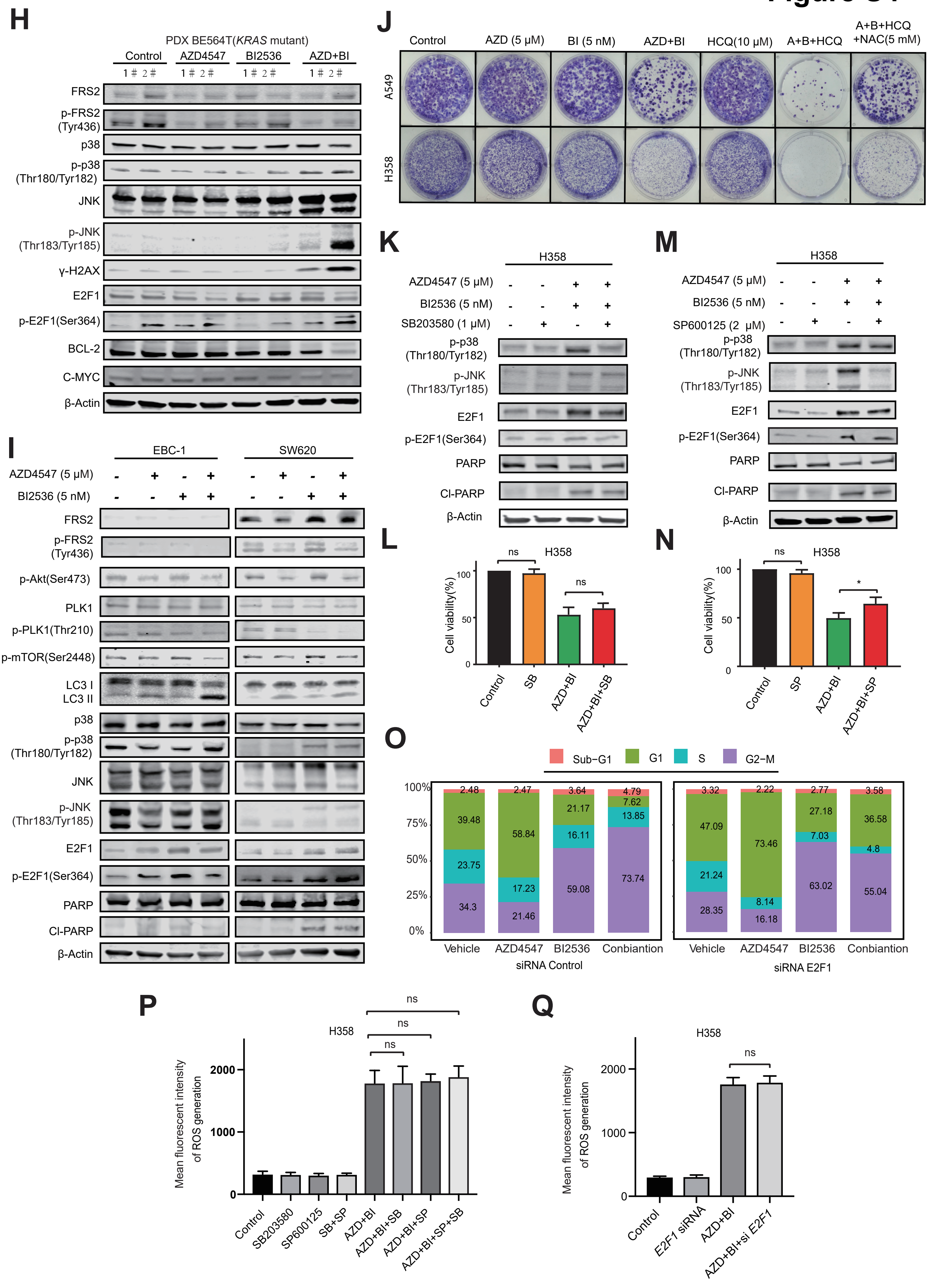
H
PDX BE564T(KRAS mutant)
Control
AZD4547
BI2536
AZD+BI
1# 2#
FRS2
p-FRS2 (Tyr436)
p38
p-p38 (Thr180/Tyr182)
JNK
p-JNK (Thr183/Tyr185)
γ-H2AX
E2F1
p-E2F1(Ser364)
BCL-2
C-MYC
β-Actin
I
EBC-1
SW620
AZD4547 (5 μM)
BI2536 (5 nM)
FRS2
p-FRS2 (Tyr436)
p-Akt(Ser473)
PLK1
p-PLK1(Thr210)
p-mTOR(Ser2448)
LC3 I
LC3 II
p38
p-p38 (Thr180/Tyr182)
JNK
p-JNK (Thr183/Tyr185)
E2F1
p-E2F1(Ser364)
PARP
Cl-PARP
β-Actin
J
Control
AZD (5 μM)
BI (5 nM)
AZD+BI
HCQ(10 μM)
A+B+HCQ
A+B+HCQ +NAC(5 mM)
A549
H358
K
H358
AZD4547 (5 μM)
BI2536 (5 nM)
SB203580 (1 μM)
p-p38 (Thr180/Tyr182)
p-JNK (Thr183/Tyr185)
E2F1
p-E2F1(Ser364)
PARP
Cl-PARP
β-Actin
M
H358
AZD4547 (5 μM)
BI2536 (5 nM)
SP600125 (2 μM)
p-p38 (Thr180/Tyr182)
p-JNK (Thr183/Tyr185)
E2F1
p-E2F1(Ser364)
PARP
Cl-PARP
β-Actin
L
H358
ns
ns
Cell viability(%)
100
50
0
Control
SB
AZD+BI
AZD+BI+SB
N
H358
ns
*
Cell viability(%)
100
50
0
Control
SP
AZD+BI
AZD+BI+SP
O
Sub-G1
G1
S
G2-M
100%
75%
50%
25%
0%
2.48
39.48
23.75
34.3
2.47
58.84
17.23
21.46
3.64
21.17
16.11
59.08
4.79
7.62
13.85
73.74
Vehicle
AZD4547
BI2536
Conbiantion
siRNA Control
3.32
47.09
21.24
28.35
2.22
73.46
8.14
16.18
2.77
27.18
7.03
63.02
3.58
36.58
4.8
55.04
Vehicle
AZD4547
BI2536
Conbiantion
siRNA E2F1
P
H358
ns
ns
ns
Mean fluorescent intensity of ROS generation
2000
1000
0
Control
SB203580
SP600125
SB+SP
AZD+BI
AZD+BI+SB
AZD+BI+SP
AZD+BI+SP+SB
Q
H358
ns
Mean fluorescent intensity of ROS generation
2000
1000
0
Control
E2F1 siRNA
AZD+BI
AZD+BI+si E2F1

Appendix Figure S4. Combined FGFR1/PLK1 inhibition upregulates ROS and activates JNK/p38/E2F1 axis

A, FGFR inhibition downregulates mTORC1, MYC, E2F, and G2/M gene expression signatures. Gene set enrichment analysis (GSEA) is based on the GEO dataset GSE73024, the transcriptome of H1581 and H520 cells treated with CH5183284/Debio 1347, a selective FGFR inhibitor.

B, Immunoblots of A549 and H358 cells treated for 24 hours with vehicle (DMSO) and AZD4547 of indicated concentrations.

C, Immunoblots of A549 cells transfected with control siRNAs, *FGFR1*- and *PLK1*-specific siRNAs, alone or in combination for 72 h.

D, E, BEAS-2B and BEAS-2B-KRAS cells treated with vehicle (DMSO), AZD4547 (5 µM) and BI2536 (5 nM), alone or in combination for 24 hours, were analyzed by flow cytometry for ROS levels (A). Relative ROS levels, a measure of mean fluorescence intensity, were shown in (B). Data are presented as mean ± SD (n=3). *P<0.05 and ****P<0.0001 by two-way ANOVA with Tukey's multiple comparisons test.

F, Flow cytometry-based measure of ROS in SW620 and EBC-1 cells treated for 24 h with AZD4547 (5 µM) and BI2536 (5 nM), alone or in combination. Quantification of relative ROS levels was shown in the right. Data are shown as mean ± SD. (n=3). *P<0.05, ****P<0.0001, and ns (P＞0.05) by two-way ANOVA with Tukey's multiple comparisons test.

G, Immunoblots of BEAS-2B and BEAS-2B-KRAS cells treated for 24 hours with vehicle (DMSO), AZD 4547 (5 µM) and BI2536 (5 nM), alone or in combination.

H, Immunoblots of PDX (BE 564T) tumors after treatment with the indicated drugs and the combination. The number (#1 and 2) indicates individual tumors from each treatment group.

I, Immunoblots of EBC-1 and SW620 cells treated with vehicle (DMSO), AZD 4547(5 µM) and BI2536 (5 nM), alone or in combination for 24 hours.

J, Clongenic assay of H358 and A549 cells treated with AZD4547, BI2536, NAC and HCQ alone or in combination as indicated.

K, L, H358 cells preincubated overnight with SB203580 (1 µM) were treated with vehicle (DMSO) or combined AZD4547 (5 µM)/BI2536 (5 nM) for additional 24 hours. The cells were then subjected to immunoblot (K) and viability assay (L). Data are presented as mean ± SD. (n=3). $P > 0.05$ (ns) by two-way ANOVA with Tukey's multiple comparisons test.

M, N, H358 Cells preincubated overnight with SP600125 (2 µM) were treated with vehicle (DMSO) and AZD4547 (5 µM)/BI2536 (5 nM) drug combination for 24 hours. The cells were then subjected to immunoblot (M) and viability assay (N). Data are presented as mean ± SD. (n=3). $^*P < 0.05$ and ns $P > 0.05$ by two-way ANOVA with Tukey's multiple comparisons test.

O, H358 cells transfected with *E2F1*-specific or control siRNAs were treated (48 h post transfection) with vehicle (DMSO), AZD4547 (5 µM) and BI2536 (5 nM), alone or in combination for 24h. The cell cycle distribution was analyzed by flow cytometry. Data are presented as mean of three independent experiments (n=3).

P, H358 cells preincubated overnight with SB203580 (1 µM) or SP600125 (2 µM), alone or combination, were further treated with vehicle (DMSO) or combined AZD4547 (5 µM)/BI2536 (5 nM) for 24 hours. The cells were then subjected to flow cytometry-based

measure and quantification of relative ROS levels. Data are presented as mean ± SD. (n=3), $P > 0.05$ (ns) by two-way ANOVA with Tukey's multiple comparisons test.

Q, H358 cells transfected with *E2F1*-specific or control siRNAs were treated (48 h post transfection) with vehicle (DMSO) or combined AZD4547 (5 μM)/BI2536 (5 nM) for additional 24 hours. Quantification of relative ROS levels was shown. Data are presented as mean ± SD. (n=3). $P > 0.05$ (ns) by two-way ANOVA with Tukey's multiple comparisons test.

Figure S5

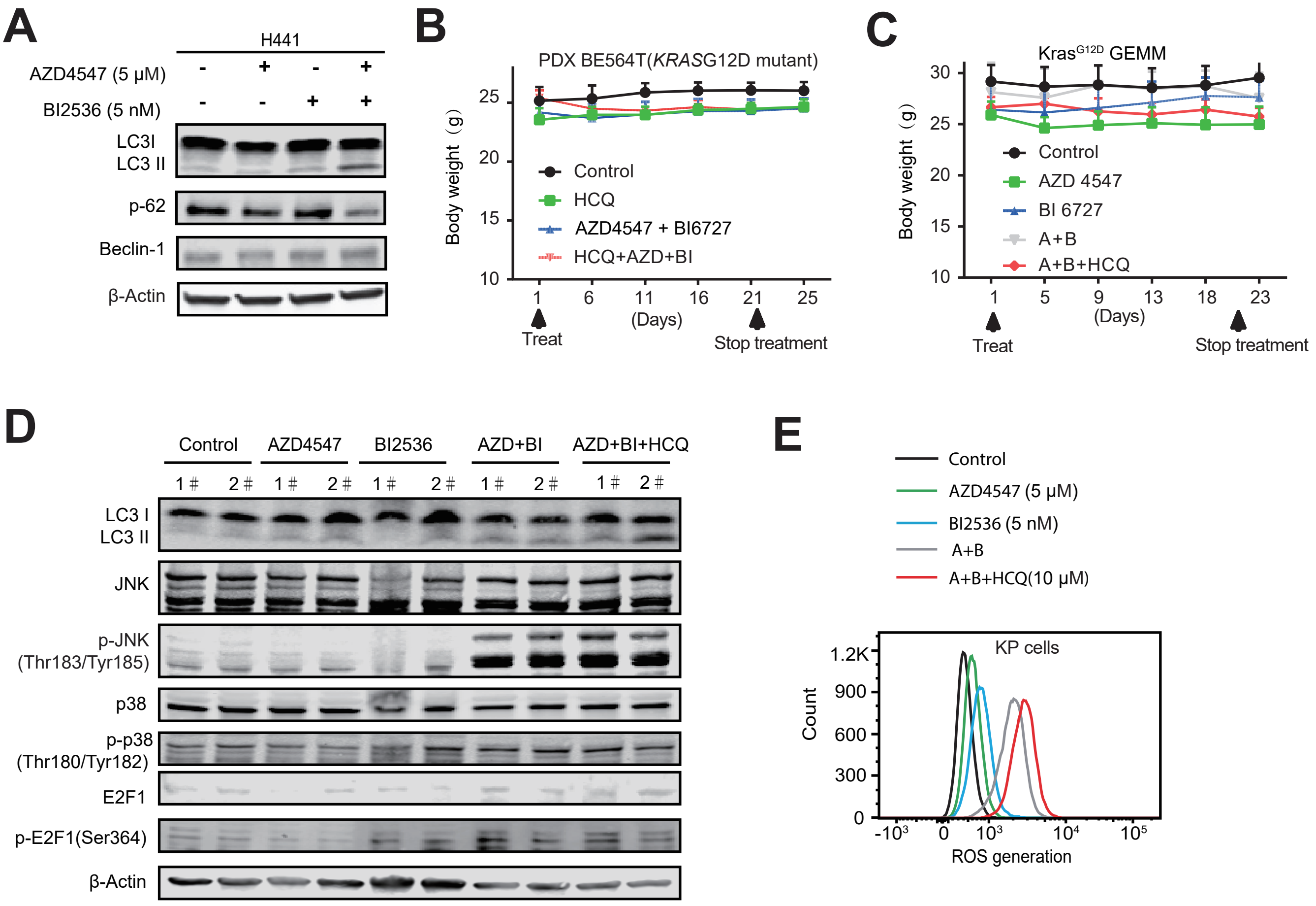
A
H441
AZD4547 (5 μM)
BI2536 (5 nM)
LC3I
LC3 II
p-62
Beclin-1
β-Actin
B
PDX BE564T(KRASG12D mutant)
Body weight（g）
Control
HCQ
AZD4547 + BI6727
HCQ+AZD+BI
(Days)
Treat
Stop treatment
C
Kras G12D GEMM
Body weight（g）
Control
AZD 4547
BI 6727
A+B
A+B+HCQ
(Days)
Treat
Stop treatment
D
Control
AZD4547
BI2536
AZD+BI
AZD+BI+HCQ
LC3 I
LC3 II
JNK
p-JNK
(Thr183/Tyr185)
p38
p-p38
(Thr180/Tyr182)
E2F1
p-E2F1(Ser364)
β-Actin
E
Control
AZD4547 (5 μM)
BI2536 (5 nM)
A+B
A+B+HCQ(10 μM)
KP cells
Count
ROS generation

Appendix Figure S5. Combined treatment with FGFR1 and PLK1 inhibitors evokes autophagy *in vitro* and *In vivo*.

A, Immunoblots of H441 cells treated with vehicle (DMSO), AZD4547 (5 µM) and BI2536 (5 nM), alone or in combination for 24h.

B, Body weights of mice bearing a patient-derived xenograft (PDX; BE 564T) of *KRAS*-mutant lung cancer during the treatment. Data are mean of body weights of each group (5 mice/group); error bar: SD.

C, Body weights of LSL-KRAS^{G12D} mice during the treatment. Data are mean of body weights of each group (4 mice/group); error bar: SD.

D, Immunoblots of LSL-KRAS^{G12D} tumors after the indicated treatment. The number (#1 and 2) indicates individual tumors from each treatment group.

E, Flow cytometry-based measure of ROS in KP cells treated for 24 h with AZD4547 (5 µM), BI2536 (5 nM) and HCQ(10 µM), alone or in combination.

Appendix Table S1. Drugs used in synthetic lethal chemical screen (related to Figure 1).

**Pathway**	**Drugs**	**Target**	**IC50 (μM) in BEAS-2B-KRAS**	**IC50 (μM) in BEAS-2B**
**RTK**	R428	AXL	6.75	6.15
	Ponatinib	RTKs	0.91	0.52
	Afatinib	EGFR	8.57	4.57
	AZD4547	FGFR1-3	6.14	7.35
**PI3K/AKT/mTOR**	AZD5363	AKT1-3	8.59	6.13
	NVP-BEZ235	PI3K/mTOR	4.45	4.56
	Rapamycin	mTOR	9.85	9.64
**RAF/MEK/ERK**	Trametinb	MEK1/2	0.24	0.11
	Sorafenib	RAF	10.28	6.17
**Synthetic Lethality**	QNZ(EVP4593)	NF-κB	25.64	24.3
	Onalespib	HSP-90	0.46	0.21
	Palbociclib	CDK4/6	15.61	16.36
	ABT-737	Bcl-2/Bcl-xL	3.73	4.13
	MG132	proteasome	10.32	13.54
	Fasudil	ROCK	146.43	143.84
	BI2536	PLK1	0.032	0.035
**Others**	SNS-314	AuroraA/B/C	1.64	1.77
	Hydroxychloroquine (HCQ)	TLR9/Autophagy	63.15	58.13
	MK-1775	WEE1	10.6	8.75
	GDC-0575	Chk1	0.48	0.53
	RGF-966	HDAC3	30.53	29.46
	Olapanib	PARP1/2	52.13	45.49

Appendix Table S2. IC_{50} values of BI2536 in cell lines (related to Figure 2).

Cancer type	Cell lines	BI2536 [IC_{50} (nM)]
KRAS MUT lung	BEAS-2B KRAS	8.27
	H2009	10.37
	A549	12.19
	H358	11.87
	H2122	10.57
	H23	12.55
	H441	9.72
	Calu-1	13.93
	Calu-6	15.74
	H460	19.84
KRAS MUT pancreas	MIA PaCa-2	8.42
	AsPC-1	16.87
	SU86.86	19.17
	PANC-1	17.88
KRAS MUT colon	LS174T	15.29
	DLD-1	8.49
	SW-620	4.59
KRAS WT cancer	PC-9	27.55
	EBC-1	23.75
	H1299	17.61
	H2405	19.84
	H1993	25.77
	H3122	4.19
	H1650	28.74
	H2228	14.88
	H28	57.49
	H2452	7.51
	JL-1	13.86
	MESO-1	19.77
	BEAS-2B	18.19
	HFBN	59.7

Appendix Table S3: Cell lines used in this study.

Cell line	Tissue	***KRAS***	***NRAS***	***c-MET***	***EGFR***	***ALK***	***BRAF***	***TP53***
H2009	Lung	MUT	WT	WT	WT	WT	WT	MUT
A549	Lung	MUT	WT	WT	WT	WT	WT	WT
H358	Lung	MUT	WT	WT	WT	WT	WT	WT
H2122	Lung	MUT	WT	WT	WT	WT	WT	MUT
H23	Lung	MUT	WT	WT	WT	WT	WT	MUT
H441	Lung	MUT	WT	WT	WT	WT	WT	MUT
Calu-1	Lung	MUT	WT	WT	WT	WT	WT	WT
Calu-6	Lung	MUT	WT	WT	WT	WT	WT	MUT
H460	Lung	MUT	WT	WT	WT	WT	WT	MUT
PF139	Lung	MUT	WT	WT	WT	WT	WT	MUT
PF563	Lung	MUT	WT	WT	WT	WT	WT	MUT
MIA PaCa-2	Pancreas	MUT	WT	WT	WT	WT	WT	MUT
AsPC-1	Pancreas	MUT	WT	WT	WT	WT	WT	MUT
SU86.86	Pancreas	MUT	WT	WT	WT	WT	WT	MUT
PANC-1	Pancreas	MUT	WT	WT	WT	WT	WT	MUT
LS174T	Colon	MUT	WT	WT	WT	WT	WT	MUT
DLD-1	Colon	MUT	WT	WT	WT	WT	WT	MUT
SW-620	Colon	MUT	WT	WT	WT	WT	WT	MUT
EBC1	Lung	WT	WT	AMP	L858R	WT	WT	MUT
PC-9	Lung	WT	WT	WT	exon 19del	WT	WT	MUT
H1650	Lung	WT	WT	WT	exon 19del	WT	WT	MUT
H1993	Lung	WT	WT	AMP	WT	WT	WT	MUT

H2228	Lung	WT	WT	WT	MUT	EML4-ALK	WT	MUT
H1299	Lung	WT	MUT	WT	WT	WT	WT	WT
H2405	Lung	WT	WT	WT	WT	WT	MUT	MUT
H3122	Lung	WT	WT	WT	WT	EML4-ALK	WT	MUT
H28	MPM	WT	WT	WT	WT	WT	WT	MUT
H2452	MPM	WT	WT	WT	WT	WT	WT	MUT
JL-1	MPM	WT	WT	WT	WT	WT	WT	MUT
MESO-1	MPM	WT	WT	WT	WT	WT	WT	MUT
HFBN1	Normal	WT	WT	WT	WT	WT	WT	WT
BEAS-2B	Normal	WT	WT	WT	WT	WT	WT	WT

Appendix Table S4: Small-molecule inhibitors used in this study.

Drugs	Target	Clinical Phase	Company	Cat. #
AZD4547	FGFR1-3	3	Sellck Chemicals	S2801
BGJ398	FGFR1-3	3	Sellck Chemicals	S2183
BI2536	PKL1	3	Sellck Chemicals	S1109
BI6727	PLK1	3	Sellck Chemicals	S2235
SB203580	P38	NA	Sellck Chemicals	S1076
SP600125	JNK	NA	Sellck Chemicals	S1460
Acetylcysteine(NAC)	ROS	Approved	Sellck Chemicals	S1623
Hydroxychloroquine(HCQ)	Autophagy	Approved	Sellck Chemicals	S4430
Q-VD-Oph	pan-caspase	NA	Sellck Chemicals	S7311

Appendix Table S5: Primary antibodies used in this study.

Antibody (for WB)	Source	Company	Cat. #	Dilution
PLK1 (208G4)	Rabbit mAb	CST	4513S	1:1000
PLK1 (phospho T210)	Rabbit mAb	Abcam	ab155095	1:500
beta-Actin (8H10D10)	Mouse mAb	CST	3700S	1:10000
Akt (pan) (11E7)	Rabbit mAb	CST	4685S	1:1000
Phospho-Akt (Ser473) (D9E)	Rabbit mAb	CST	4060S	1:1000
Phospho-Histone H2AX (Ser139)	Rabbit mAb	CST	9718S	1:1000
PARP	Rabbit mAb	CST	9532S	1:1000
p44/42 MAPK (Erk1/2) (137F5)	Rabbit mAb	CST	4695T	1:1000
Phospho-p44/42 MAPK (Erk1/2) (Thr202/Tyr204) (197G2)	Rabbit mAb	CST	4377S	1:1000
SAPK/JNK Antibody	Rabbit mAb	CST	9252	1:1000
Phospho SAPK/JNK (Thr183/Tyr185)	Rabbit mAb	CST	9251S	1:1000
p38 MAPK(D13E1)	Rabbit mAb	CST	8690T	1:1000
Phospho-p38 MAPK (Thr180/Tyr182)	Rabbit mAb	CST	4511T	1:1000
c-Myc (D3N8F)	Rabbit mAb	CST	13987S	1:1000
phospho-E2F1(Ser 364)	Rabbit mAb	abcam	ab5391	1:1000
E2F1 Antibody	Rabbit mAb	CST	3742S	1:1000
Cleaved Caspase-3 (Asp175)	Rabbit mAb	CST	9661S	1:1000
SQSTM1/p62	Rabbit mAb	CST	5114S	1:1000
LC3A/B (D3U4C)	Rabbit mAb	CST	12741S	1:1000
Beclin-1 (D40C5)	Rabbit mAb	CST	3495S	1:1000
Atg5 (D5F5U)	Rabbit mAb	CST	12994T	1:1000
K-Ras Antibody	Rabbit mAb	CST	53270	1:1000
Ras (G12V Mutant Specific) (D2H12)	Rabbit mAb	CST	14412	1:1000
Bcl2	Mouse mAb	CST	15071	1:1000
FRS2	Mouse mAb	SANTA CRUZ	sc-17841	1:200
Phospho-FRS2(Tyr436)	Rabbit mAb	CST	3861S	1:1000
mTOR(7C10)	Rabbit mAb	CST	2983P	1:1000

Phospho-mTOR(Ser2448)	Rabbit mAb	CST	2971S	1:1000
p70 S6 Kinase(49D7)	Rabbit mAb	CST	2708P	1:1000
Phospho-p70 S6 Kinase(Thr389)	Rabbit mAb	CST	9243P	1:1000
**Antibody (for IHC)**	**Source**	**Company**	**Cat. #**	**Dilution**
Ki-67	Rabbit mAB	CST	9027	1:400
Caspase 3	Rabbit mAB	CST	9664	1:2000
