# Supplementary figures and images for: Synergistic effects of FGFR1 and PLK1 inhibitors target a metabolic liability in KRAS‐mutant cancer
Source: EMBO Mol Med. 2021 Aug 8;13(9):e13193. doi: 10.15252/emmm.202013193 (PMC8422071; doi:10.15252/emmm.202013193)

Figure 2

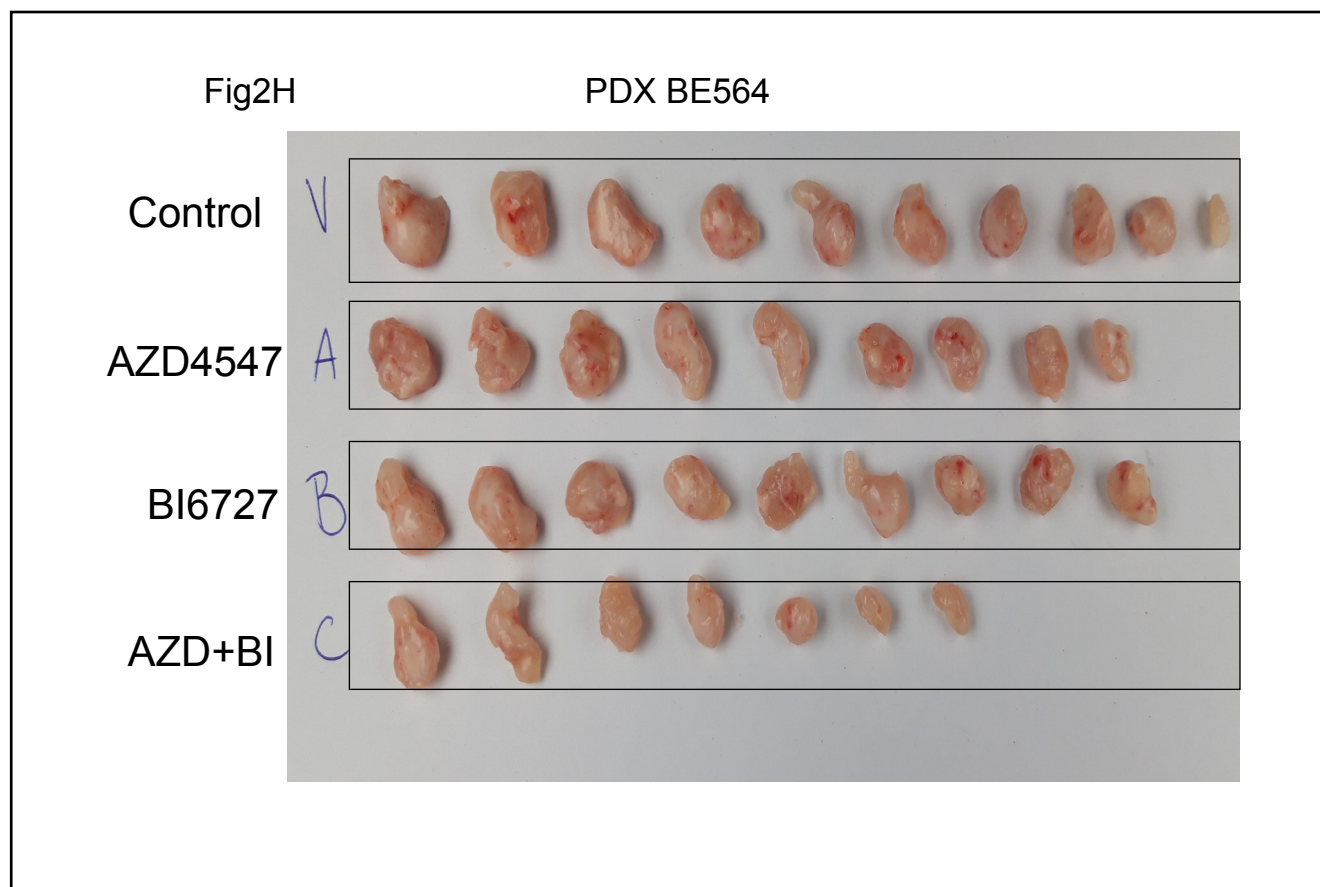

Supplement: Supplementary file 3 — Source Data for Figure 2 [file EMMM-13-e13193-s001.pdf]

Figure 3

Fig3E

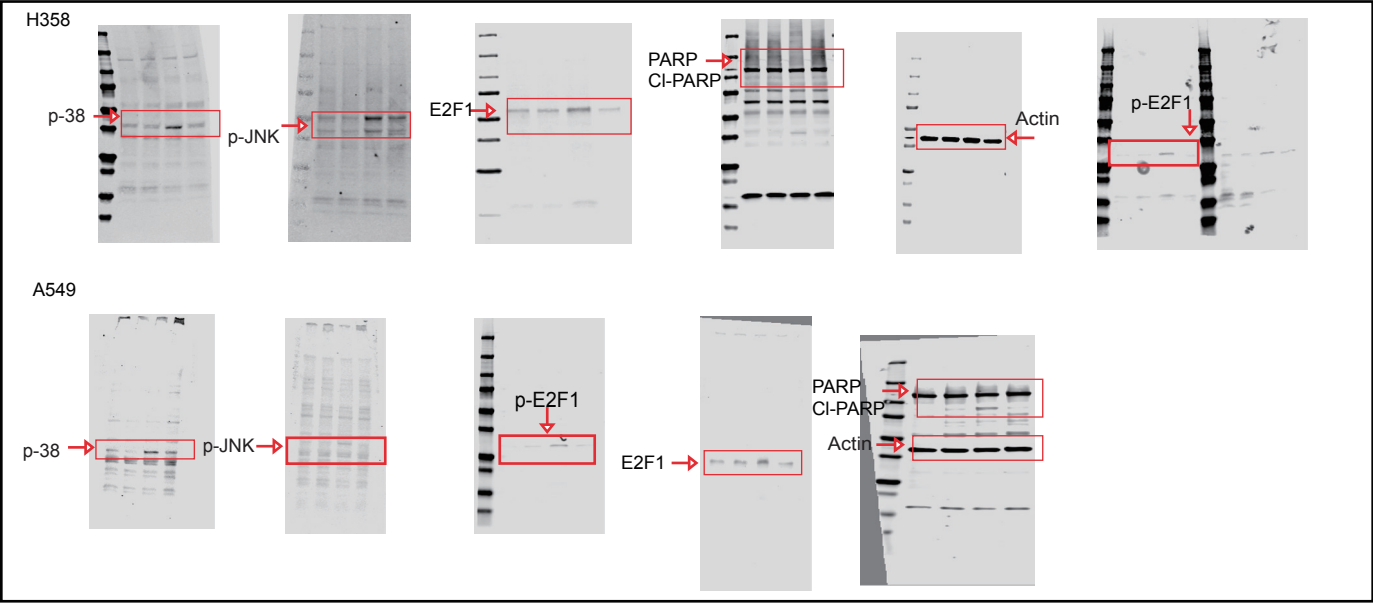

Fig3F

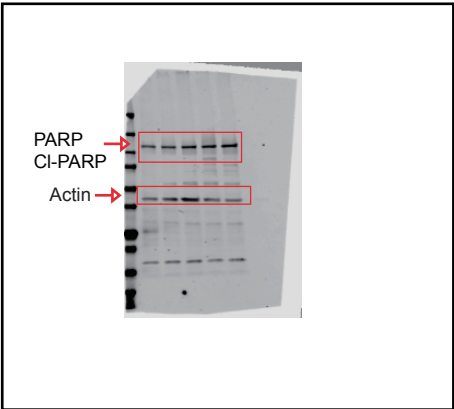

Supplement: Supplementary file 4 — Source Data for Figure 3 [file EMMM-13-e13193-s003.pdf]

Fig4A

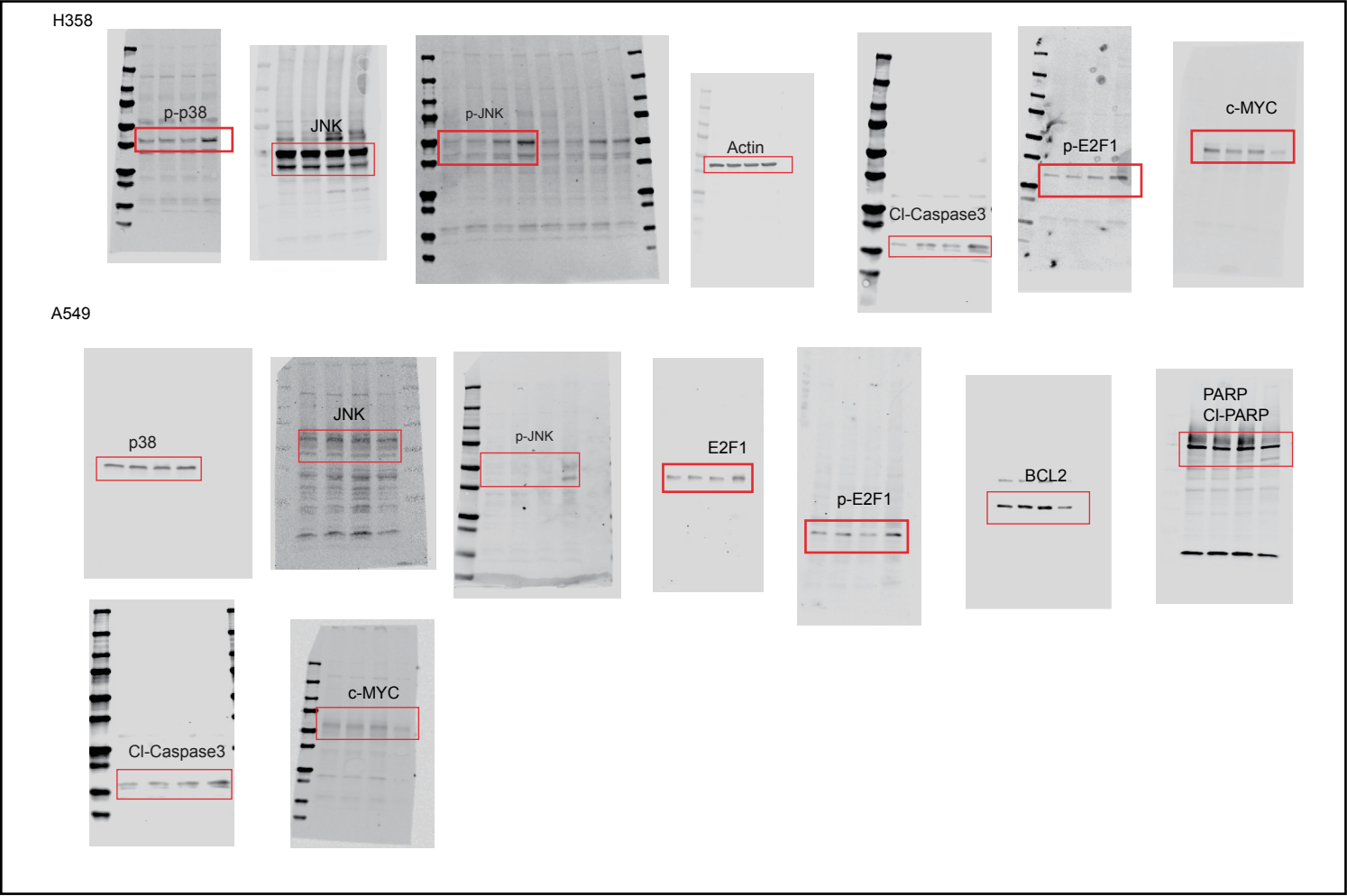

Fig4B

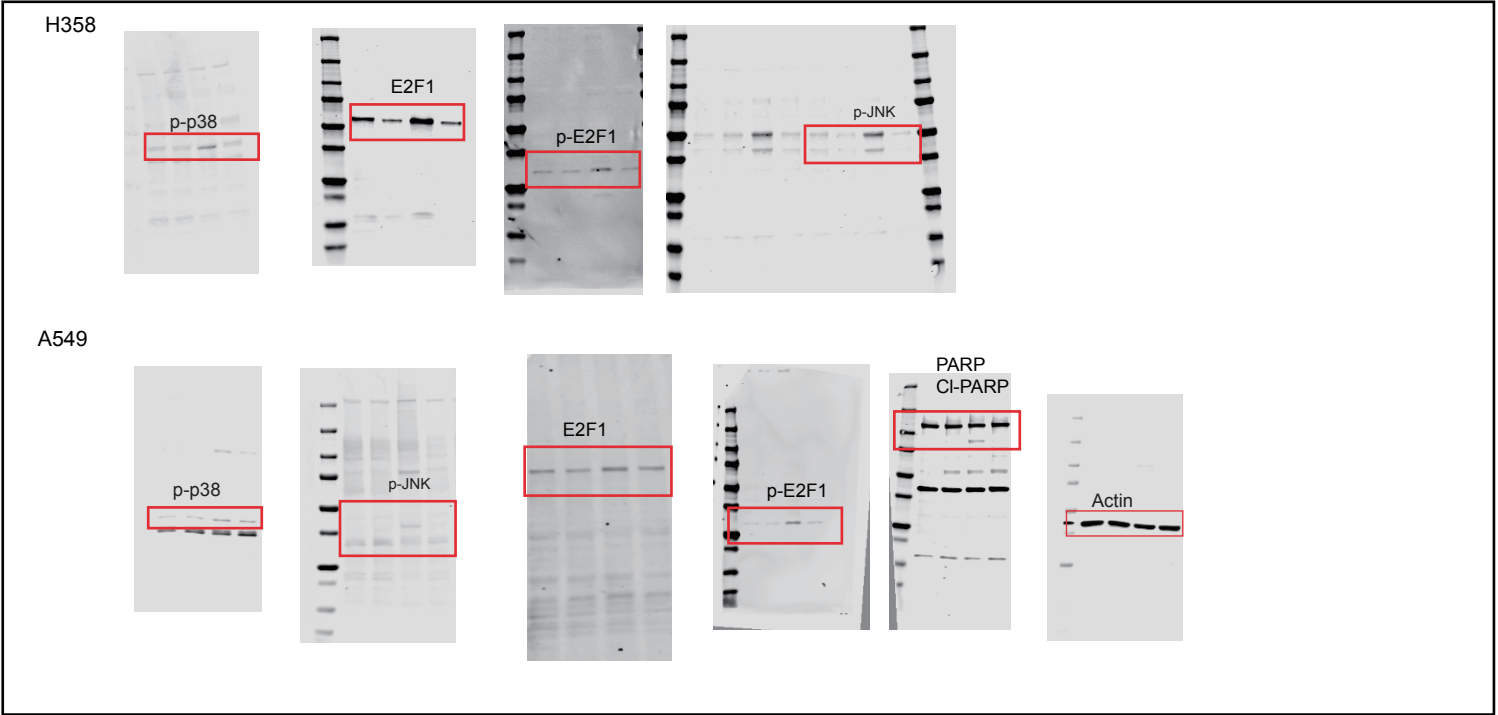

Supplement: Supplementary file 5 — Source Data for Figure 4 [file EMMM-13-e13193-s008.zip › Source data Figure 4-1.pdf]

Fig 4

Fig4D

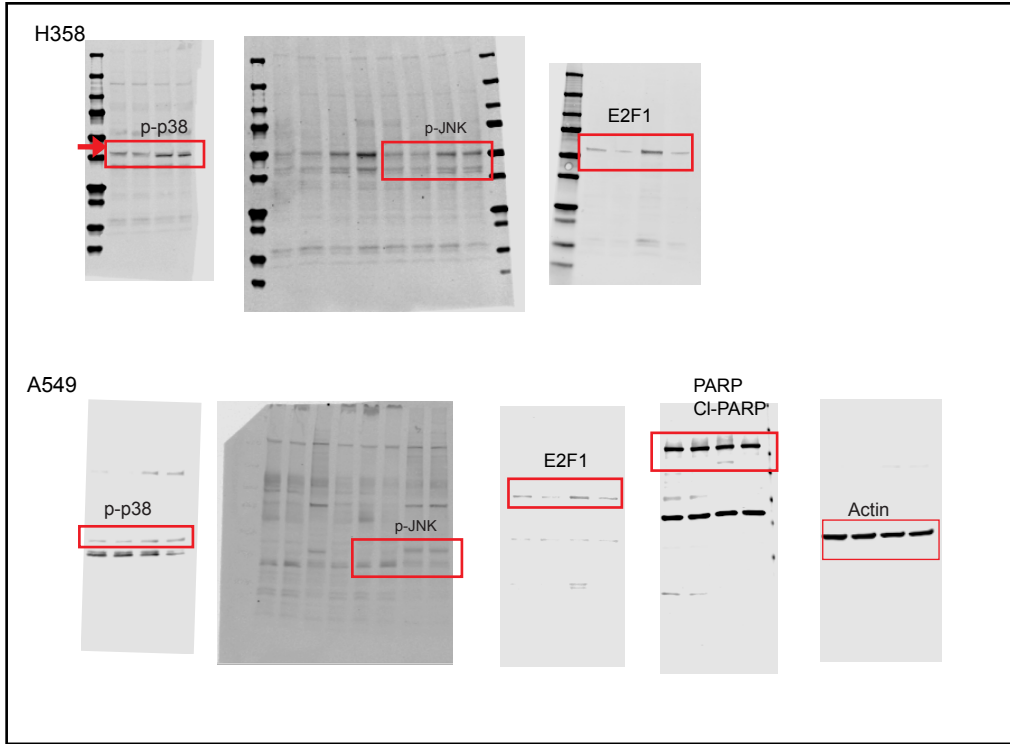

Supplement: Supplementary file 5 — Source Data for Figure 4 [file EMMM-13-e13193-s008.zip › Source data Figure 4-2.pdf]

Fig5A

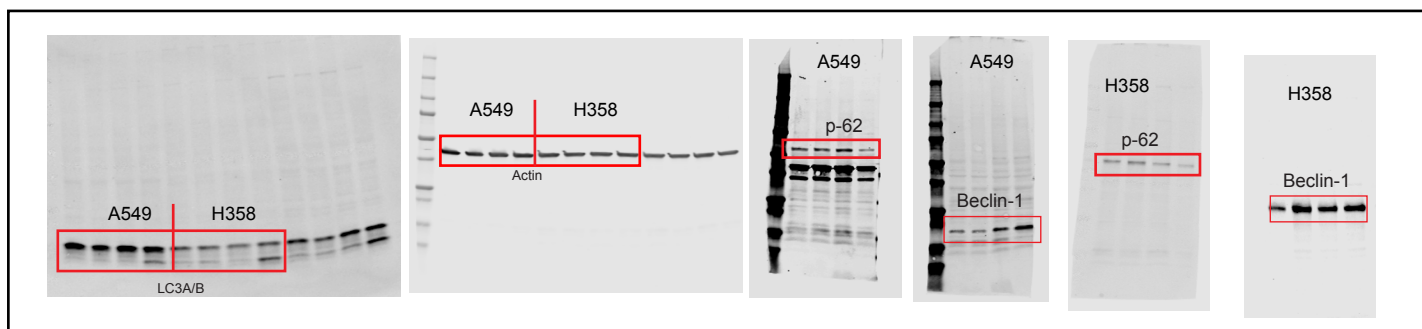

Fig5F

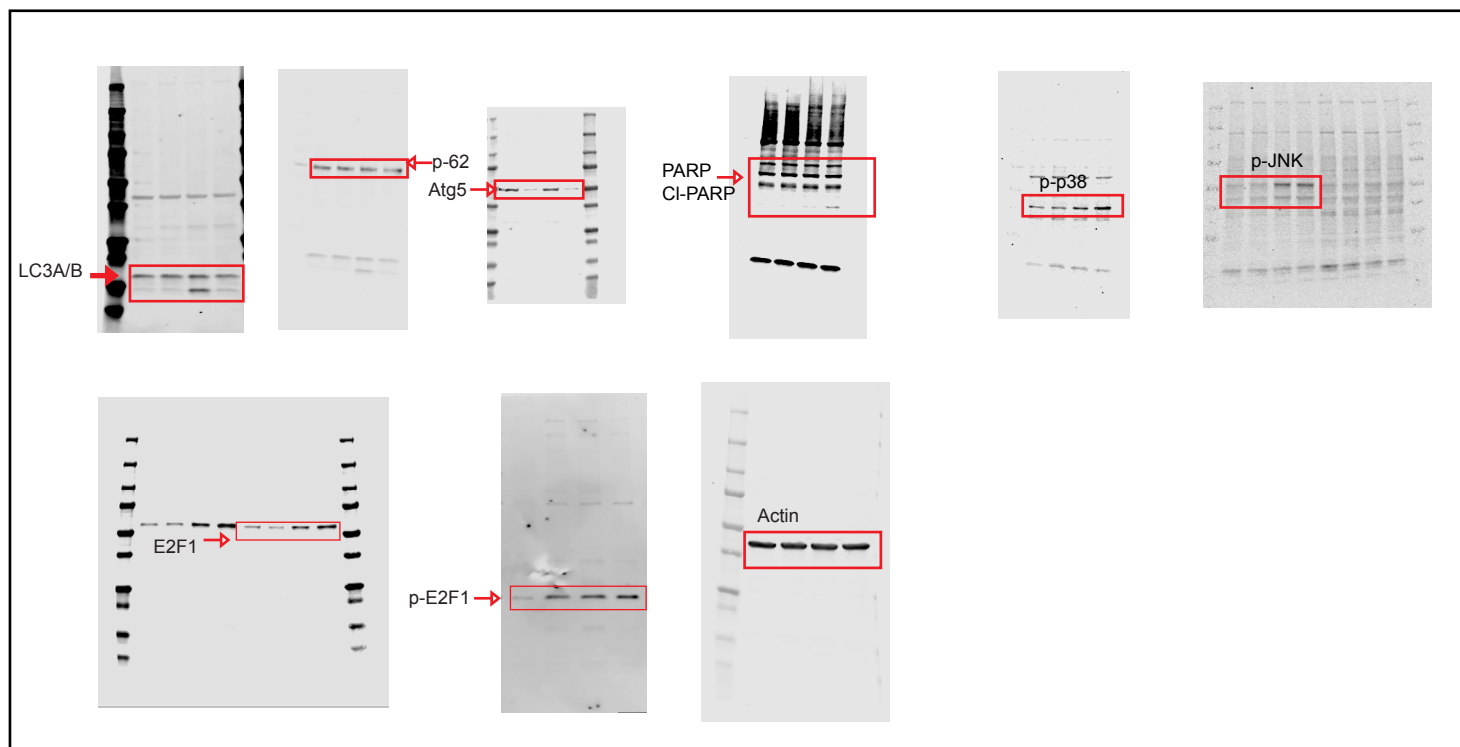

Supplement: Supplementary file 6 — Source Data for Figure 5 [file EMMM-13-e13193-s009.zip › Source data Figure 5A & 5F.pdf]

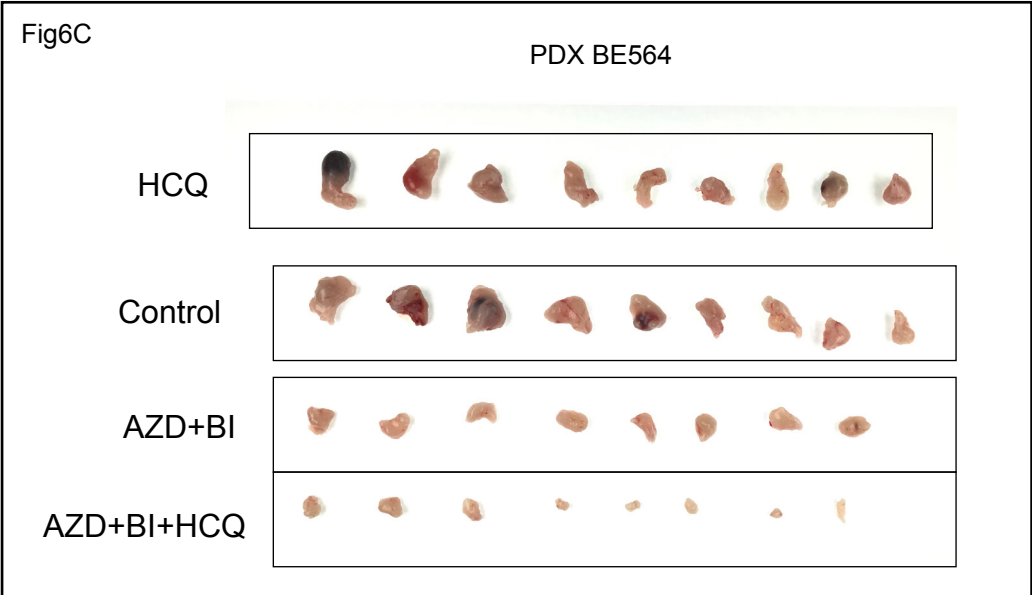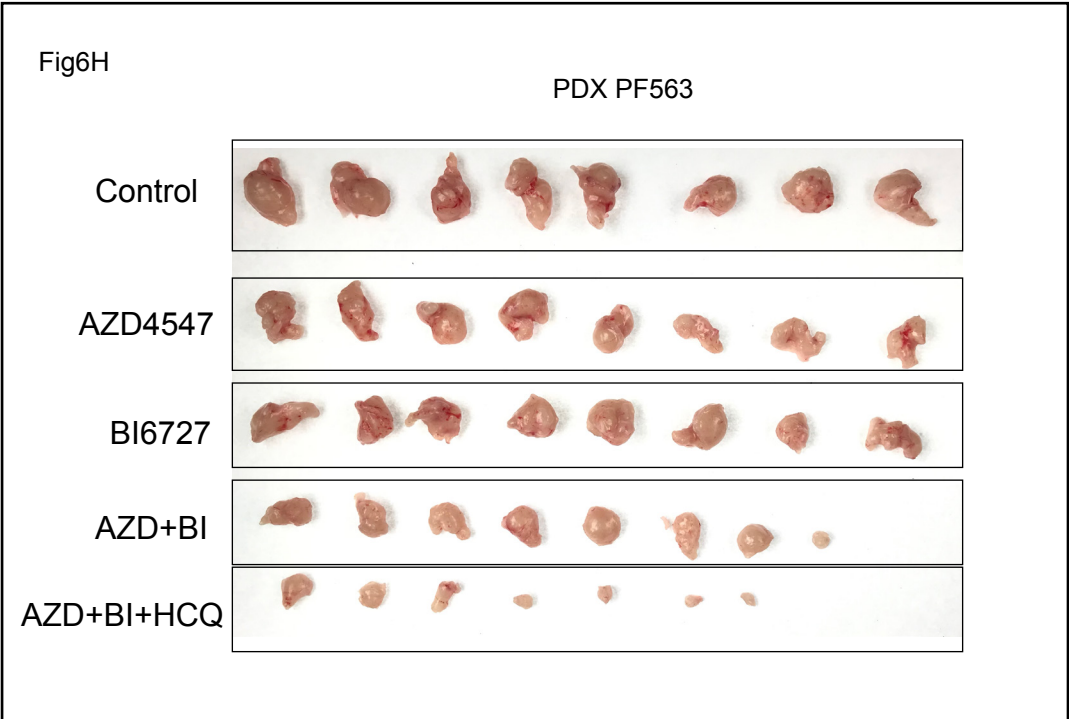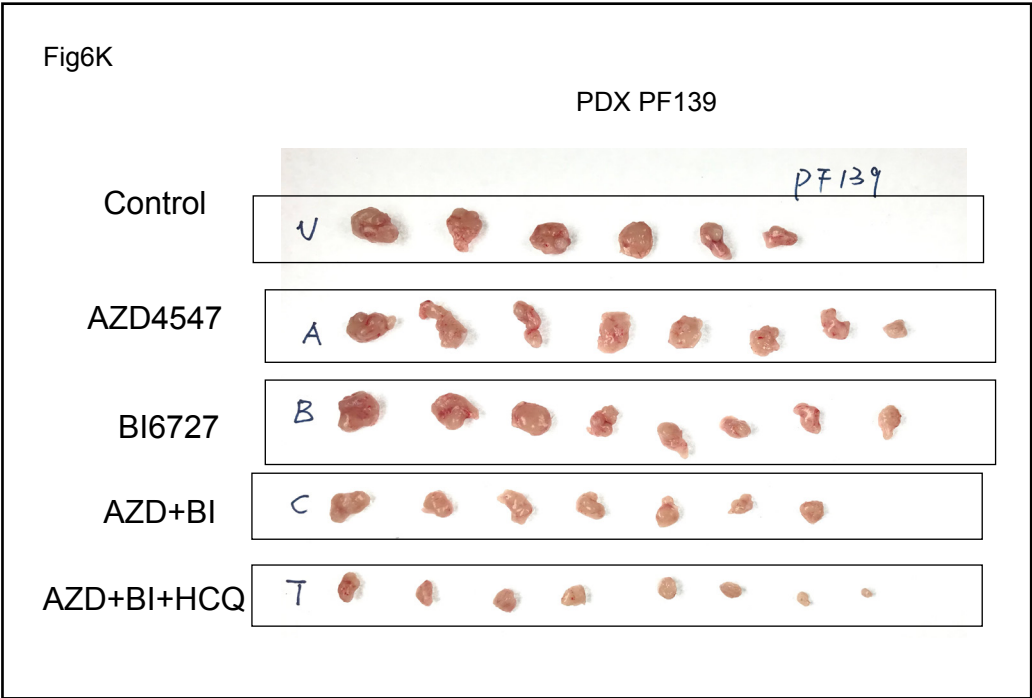

Supplement: Supplementary file 7 — Source Data for Figure 6 [file EMMM-13-e13193-s004.pdf]

Figure 7

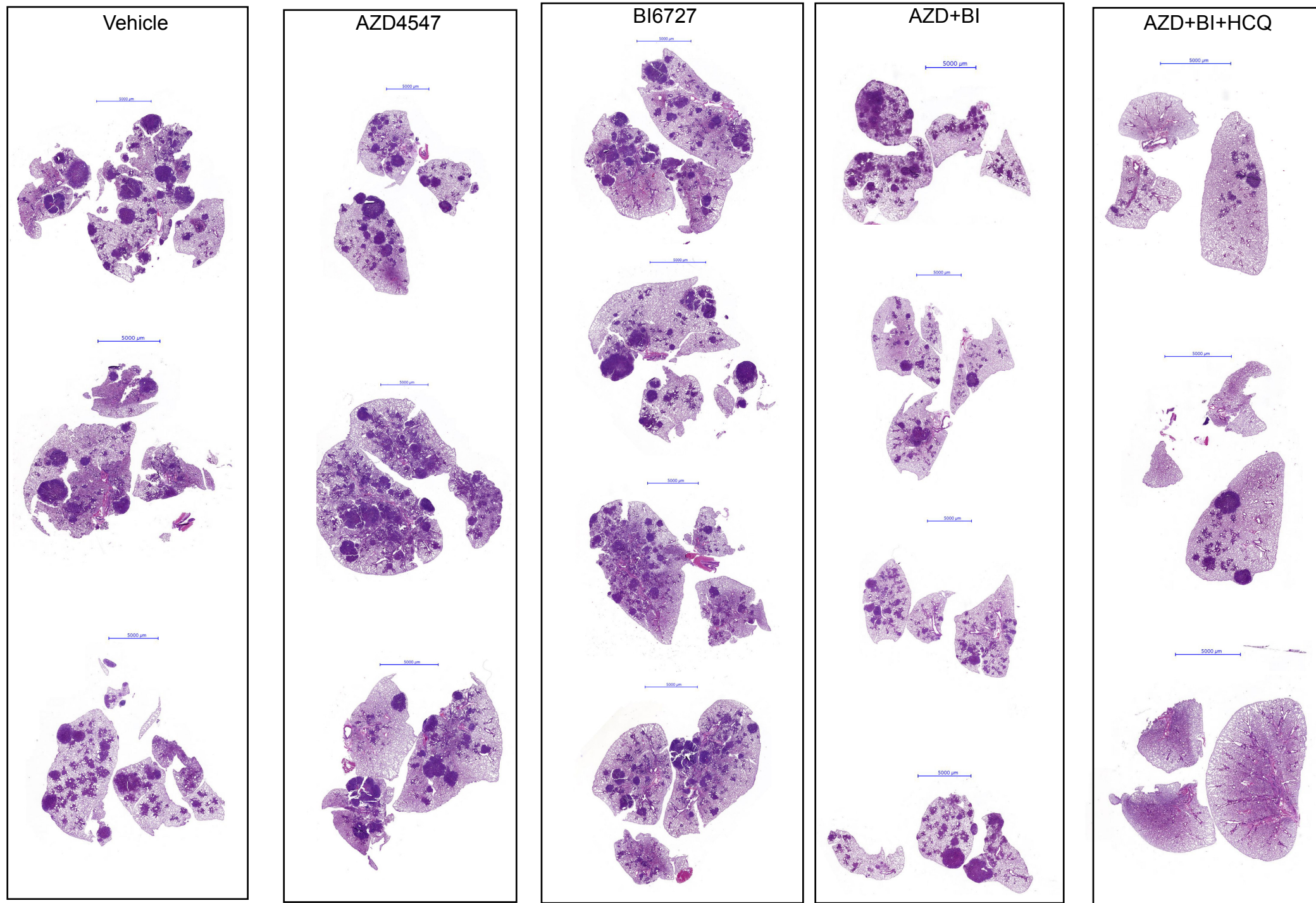

Supplement: Supplementary file 8 — Source Data for Figure 7 [file EMMM-13-e13193-s007.pdf]

Fig S5

Fig. S5A

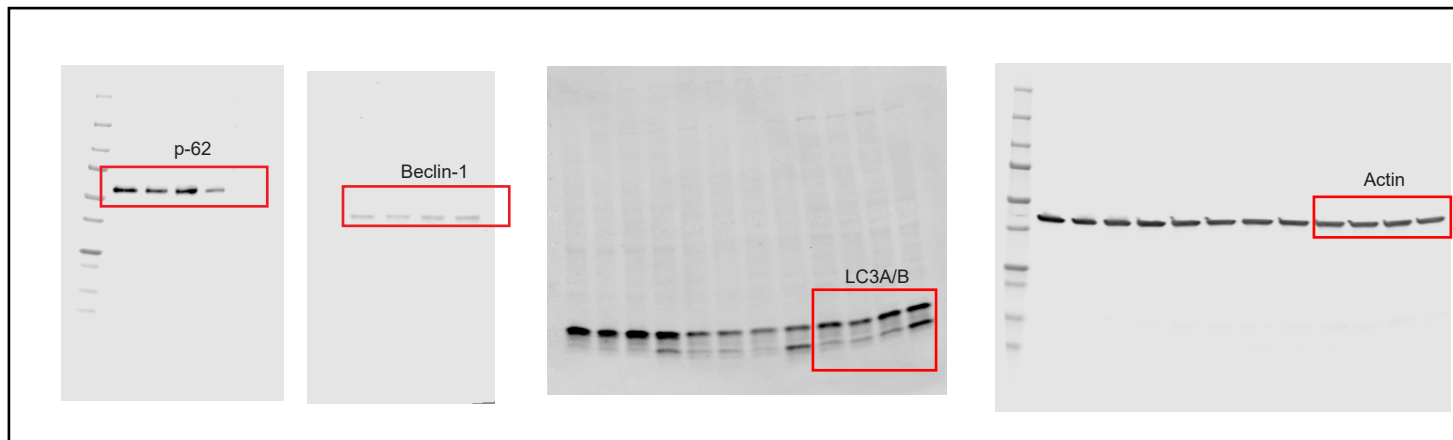

Fig S5D

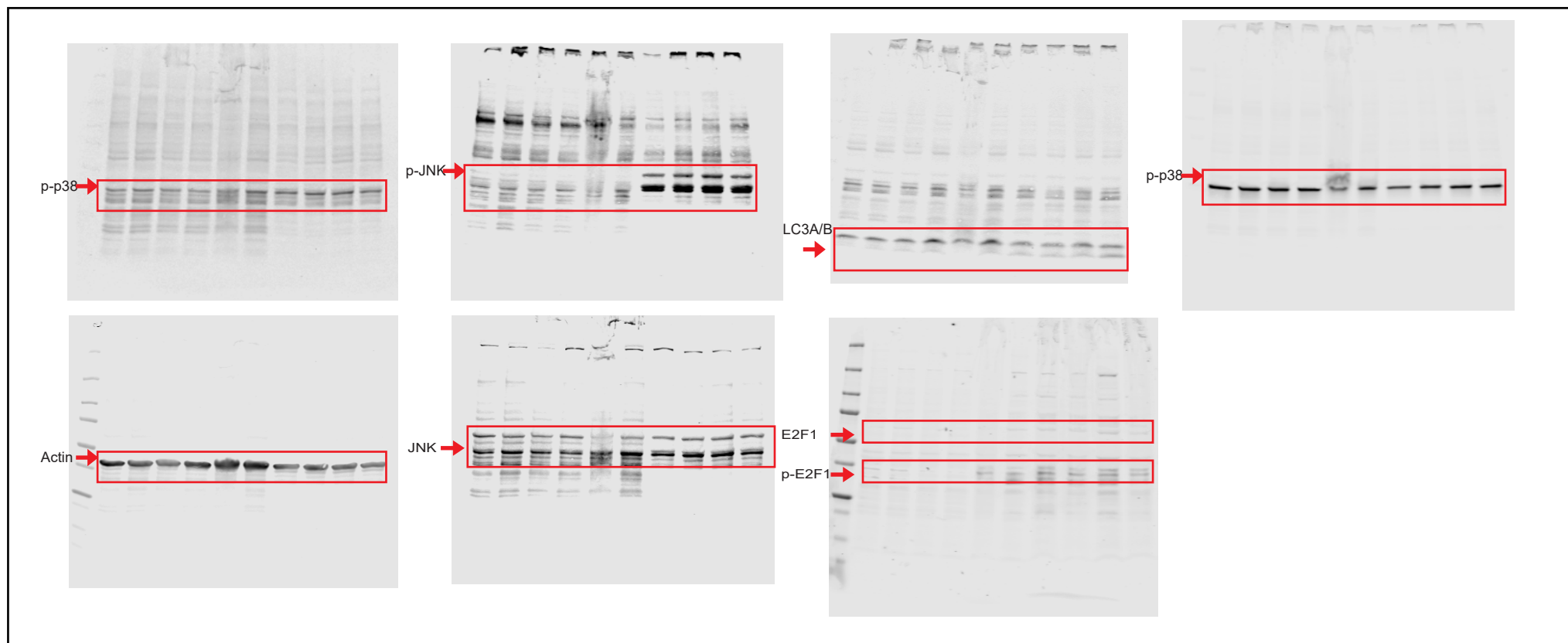

Supplement: Supplementary file 9 — Source Data for Appendix [file EMMM-13-e13193-s002.zip › Source data_Appendix Fig. S5.pdf]

Fig S2J

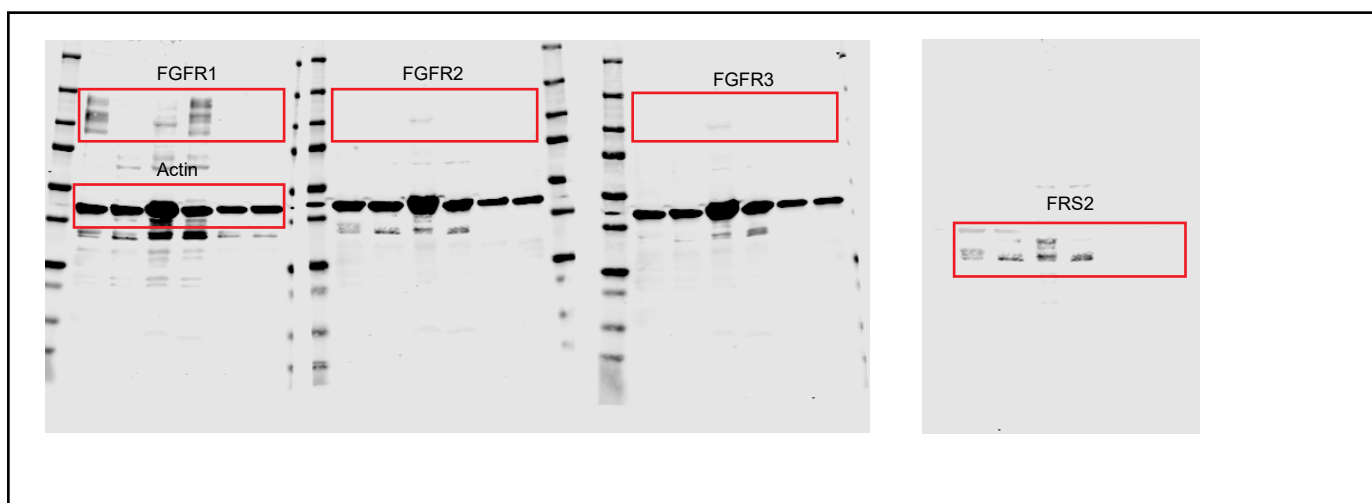

Supplement: Supplementary file 9 — Source Data for Appendix [file EMMM-13-e13193-s002.zip › Source data_Appendix Fig. S2.pdf]

Fig S1A

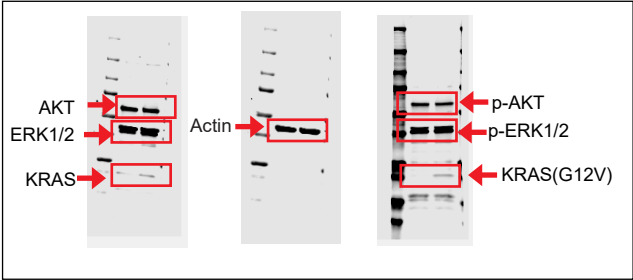

Fig. S1D

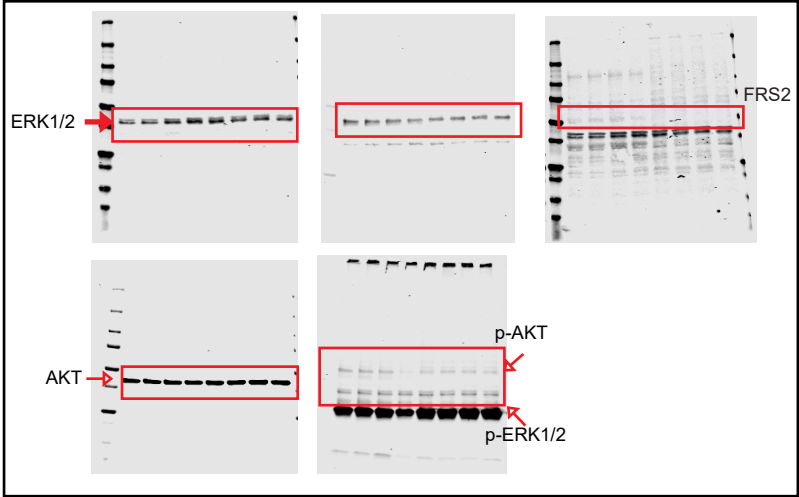

Fig S1F

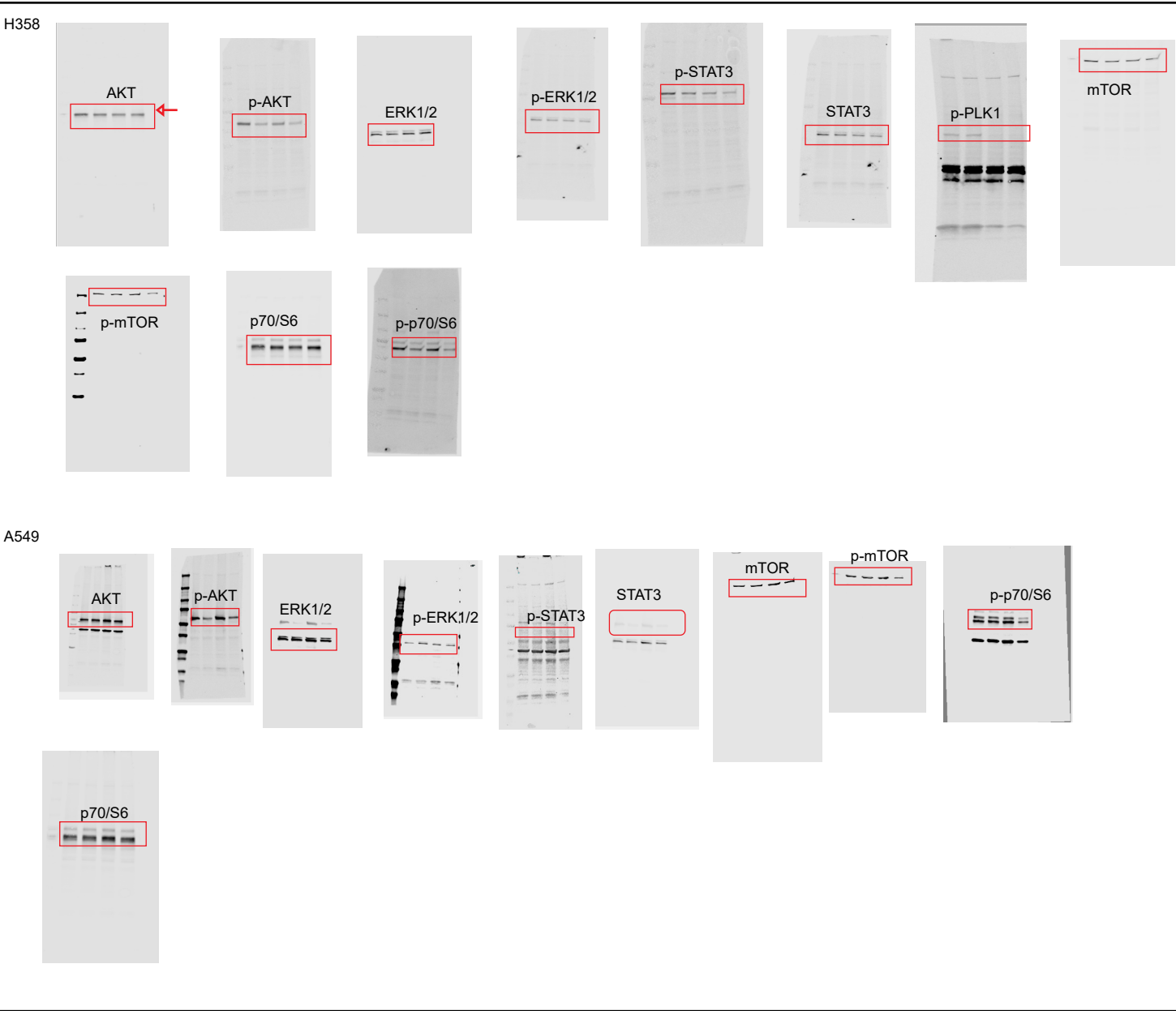

Supplement: Supplementary file 9 — Source Data for Appendix [file EMMM-13-e13193-s002.zip › Source data_Appendix Fig. S1.pdf]
