# Supplementary material for: Synergistic effects of FGFR1 and PLK1 inhibitors target a metabolic liability in KRAS‐mutant cancer
Source: EMBO Mol Med. 2021 Aug 8;13(9):e13193. doi: 10.15252/emmm.202013193 (PMC8422071; doi:10.15252/emmm.202013193)

Fig 5B:
Control DAPI

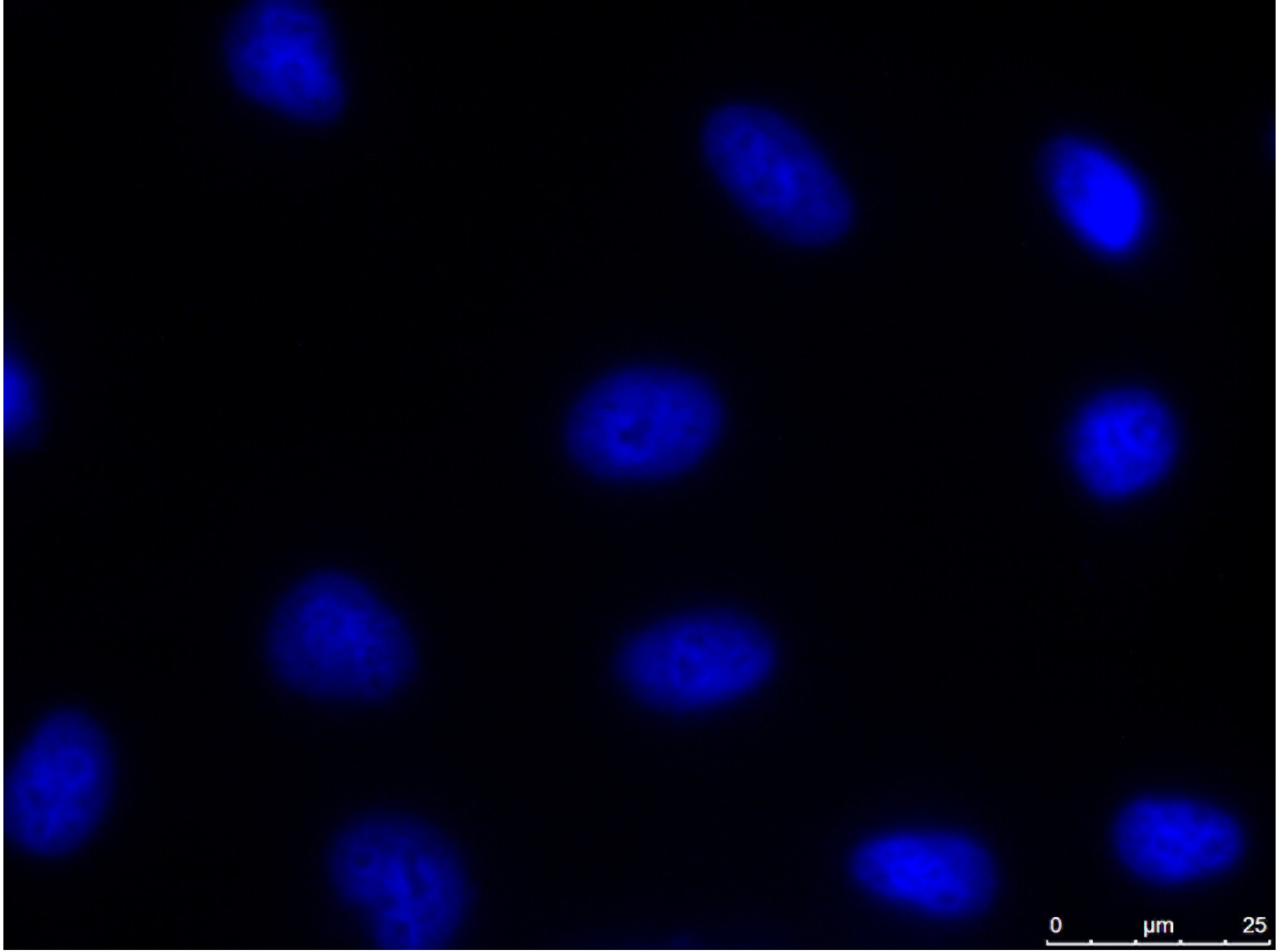

Fig 5B:
Control GFP

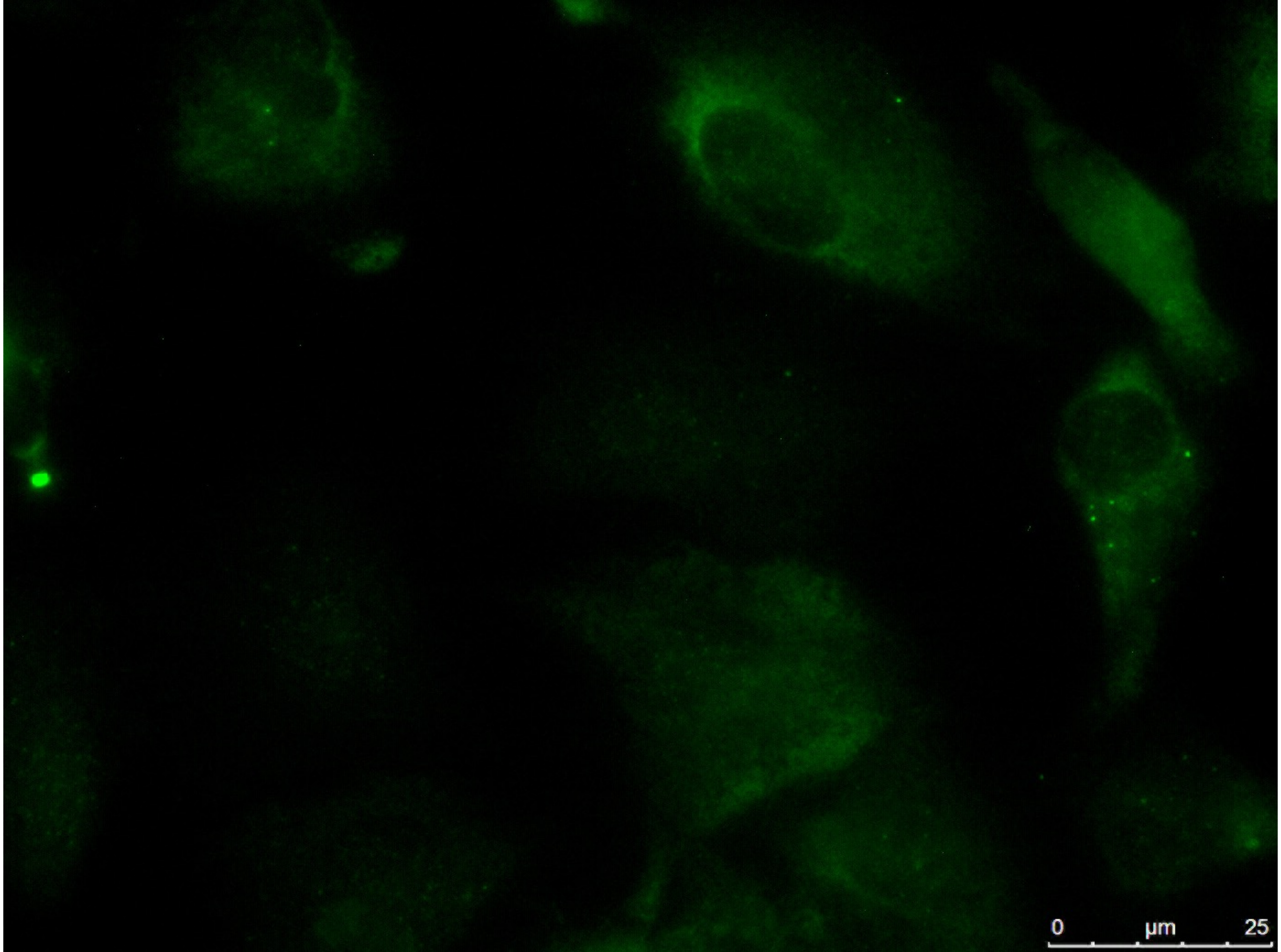

Fig 5B:
Control mCherry

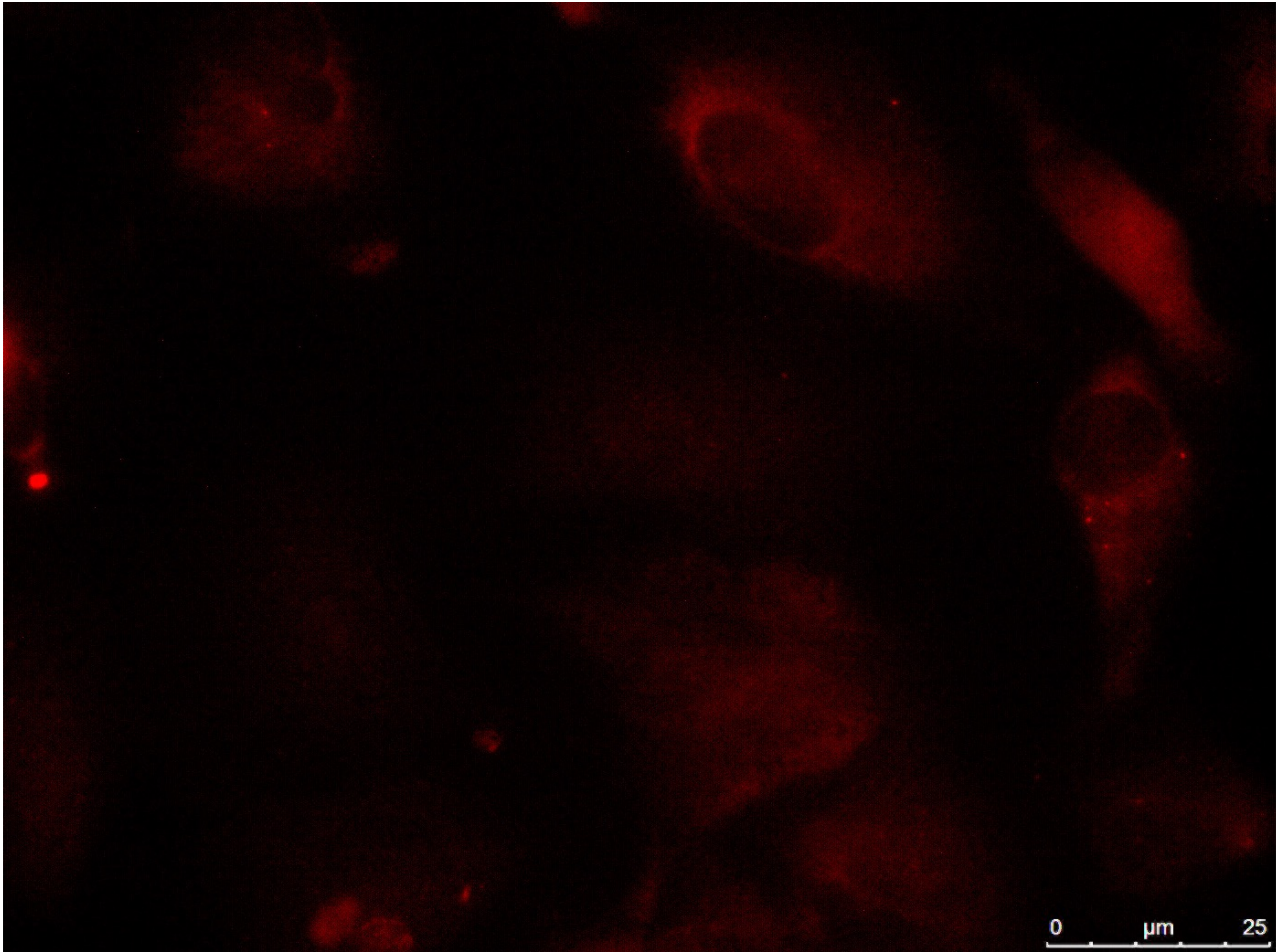

Fig 5B:
Control Merge

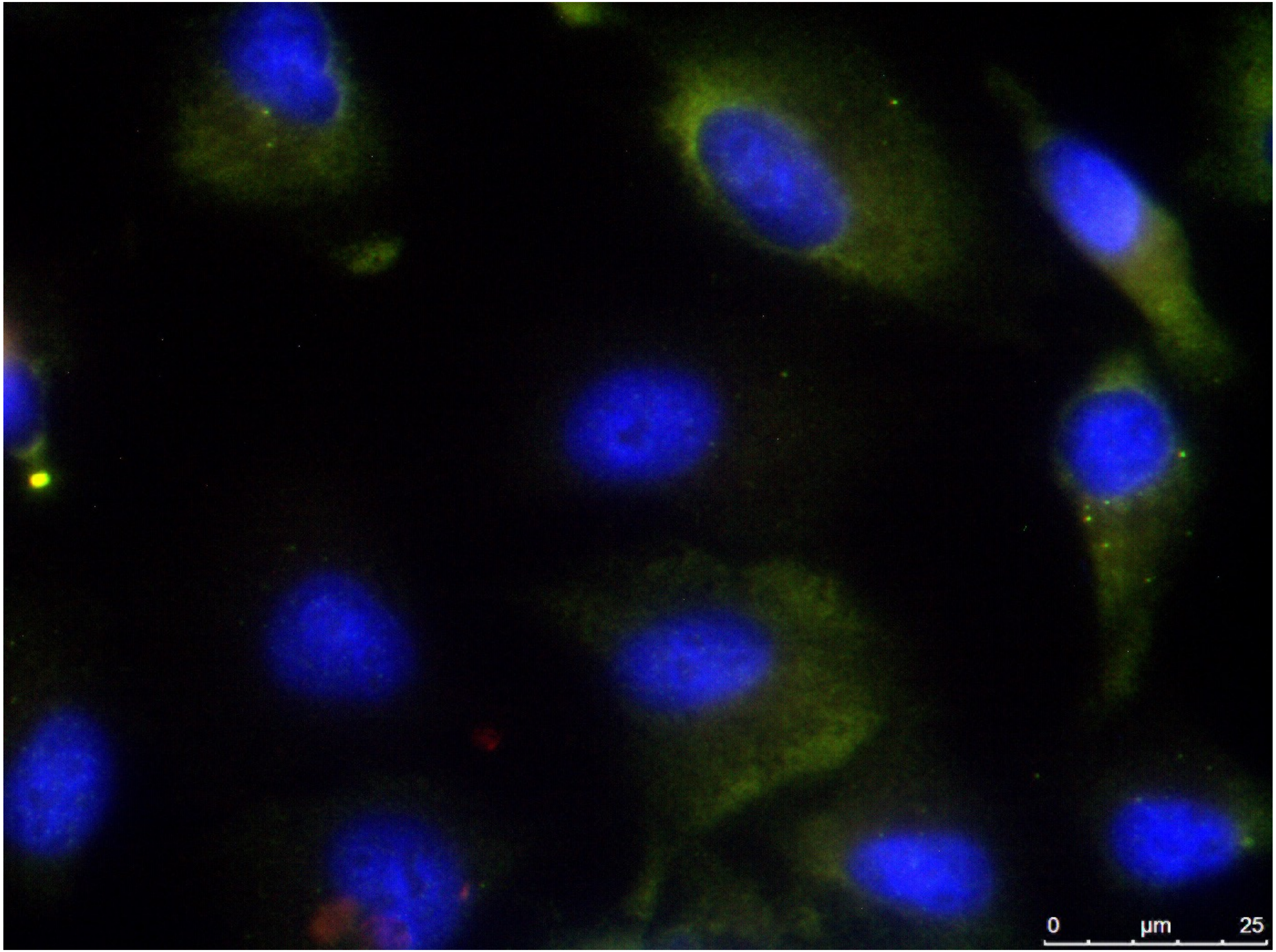

Fig 5B:
AZD4547 DAPI

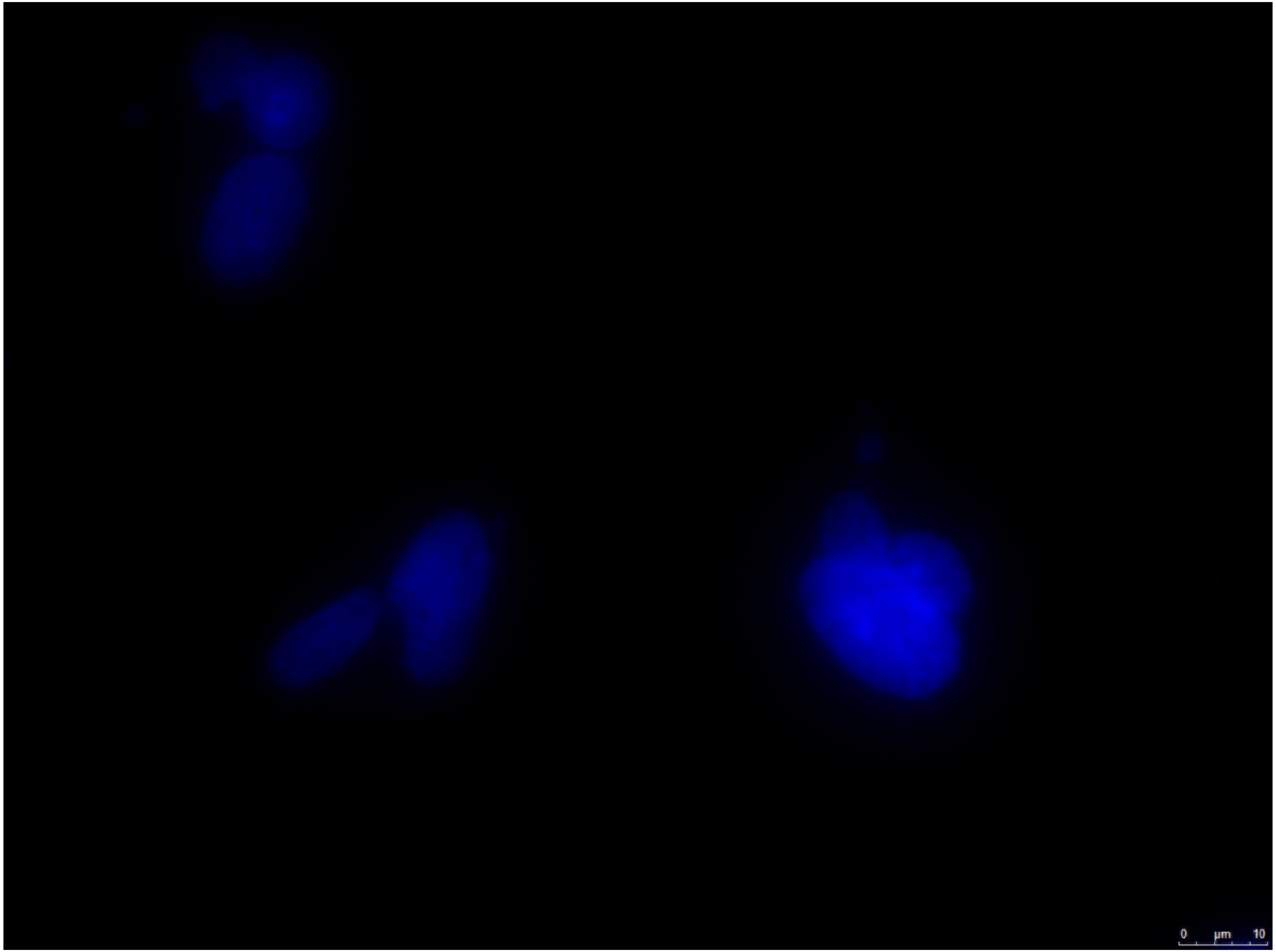

Fig 5B:
AZD4547 GFP

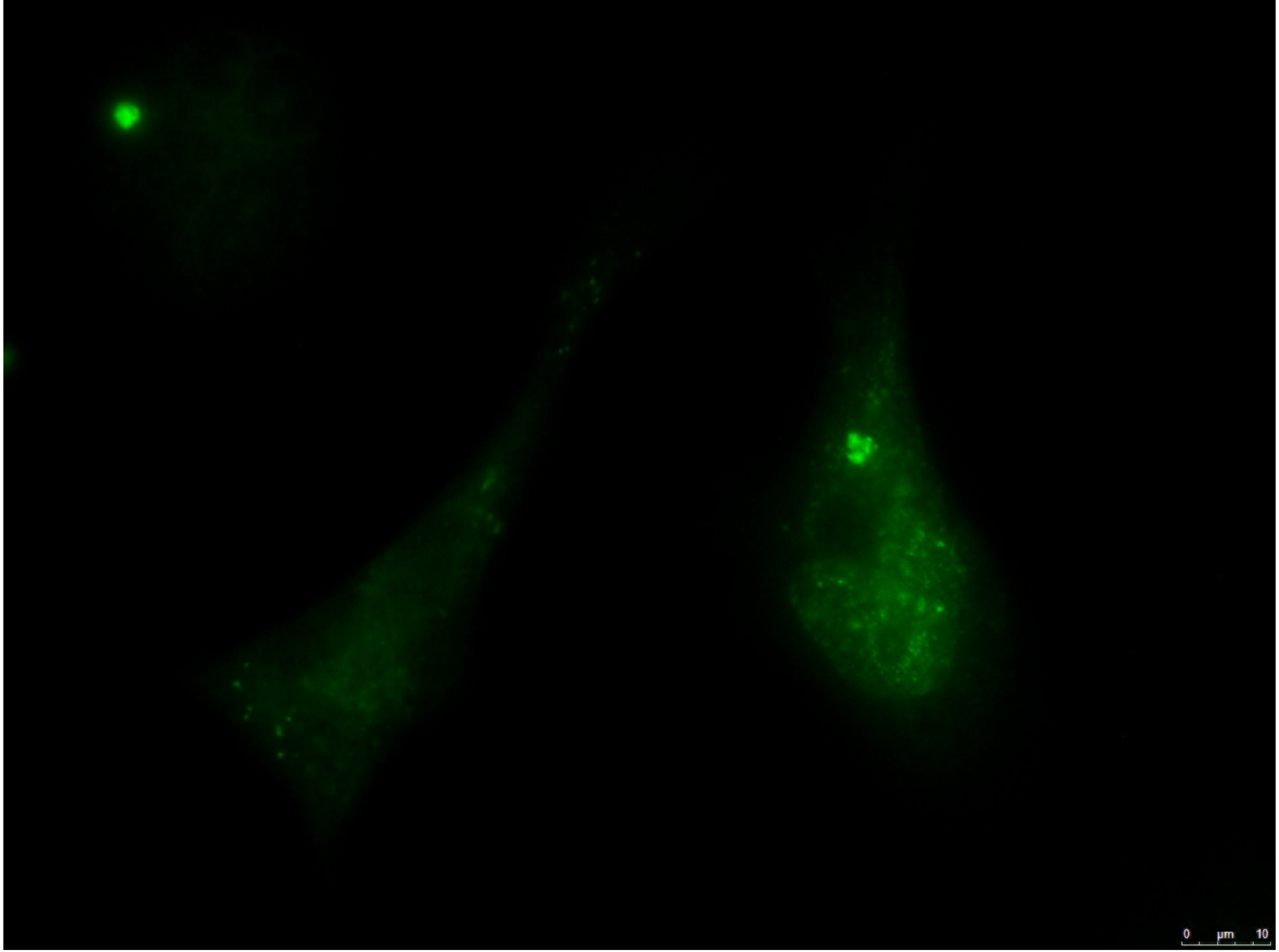

Fig 5B:
AZD4547 mCherry

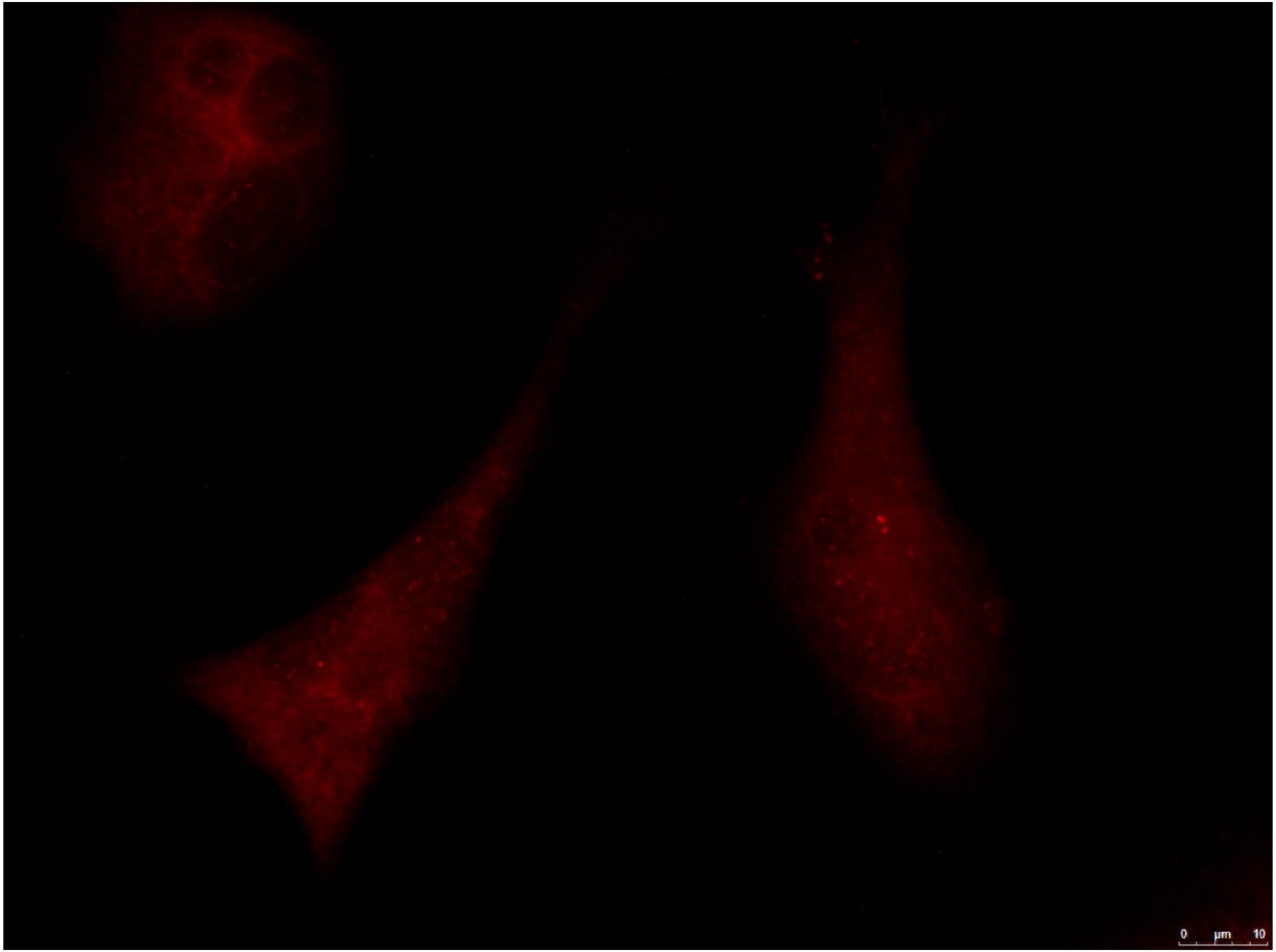

Fig 5B:
AZD4547 Merge

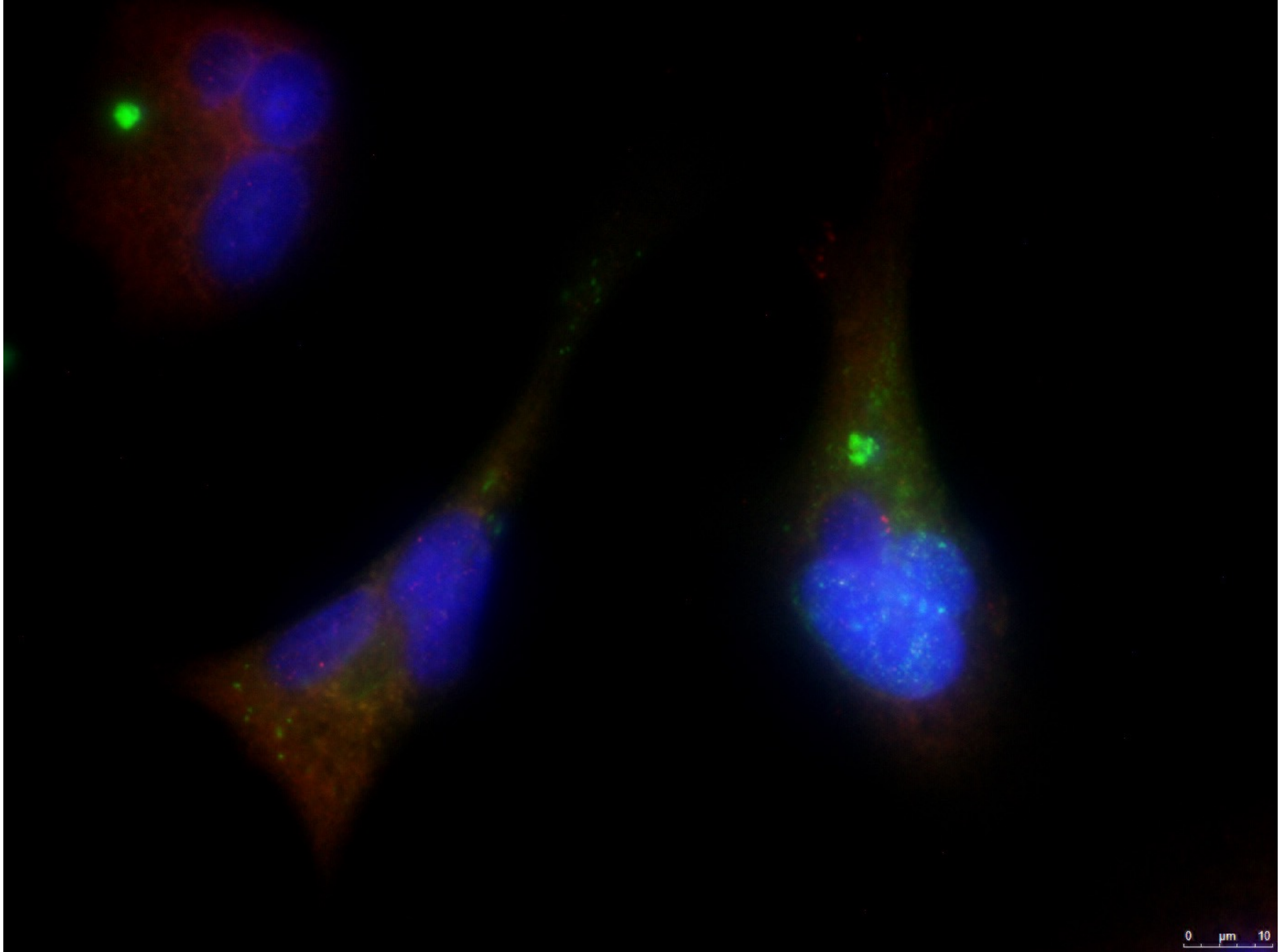

Fig 5B:
BI2536 DAPI

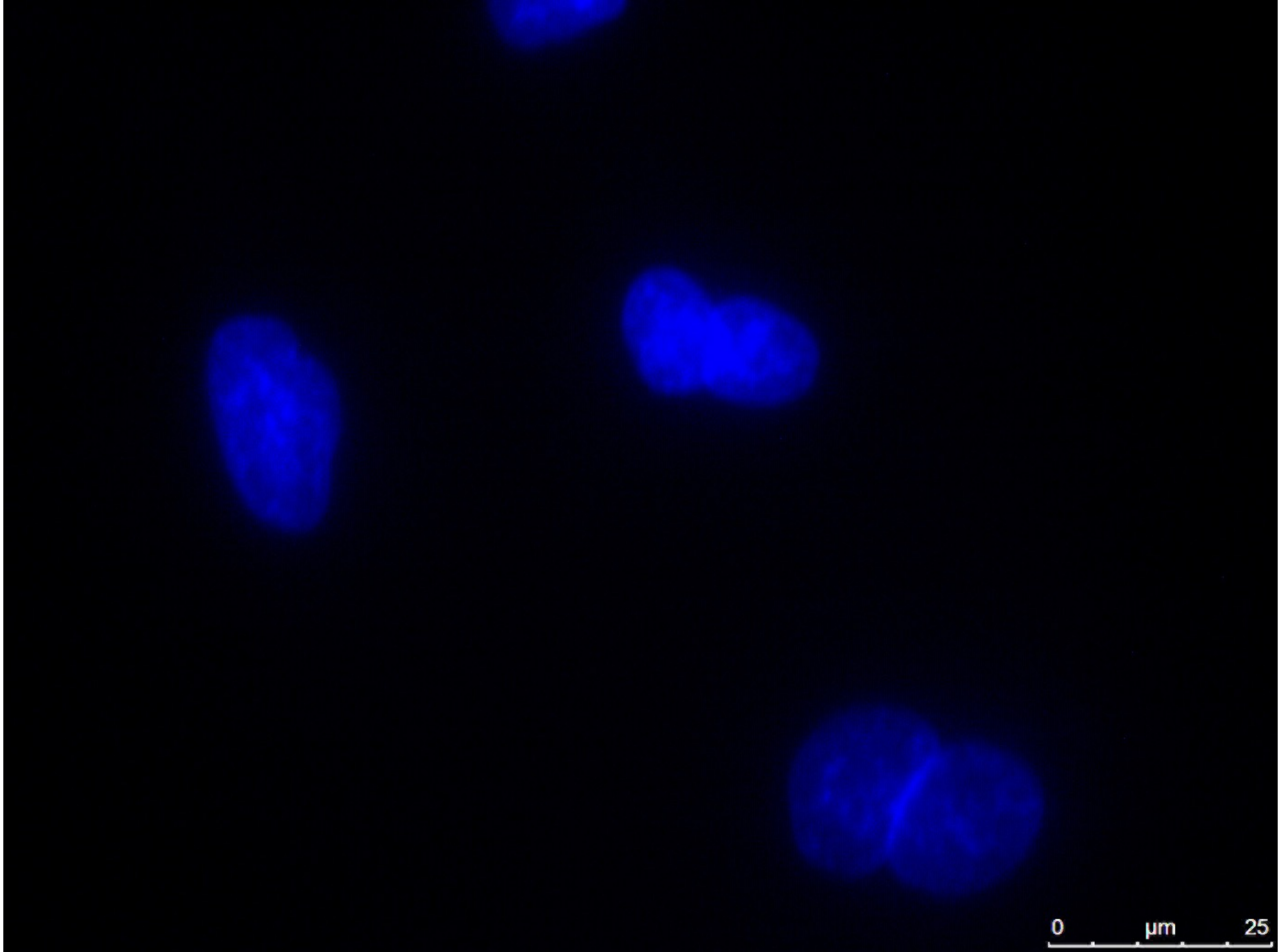

Fig 5B:
BI2536 GFP

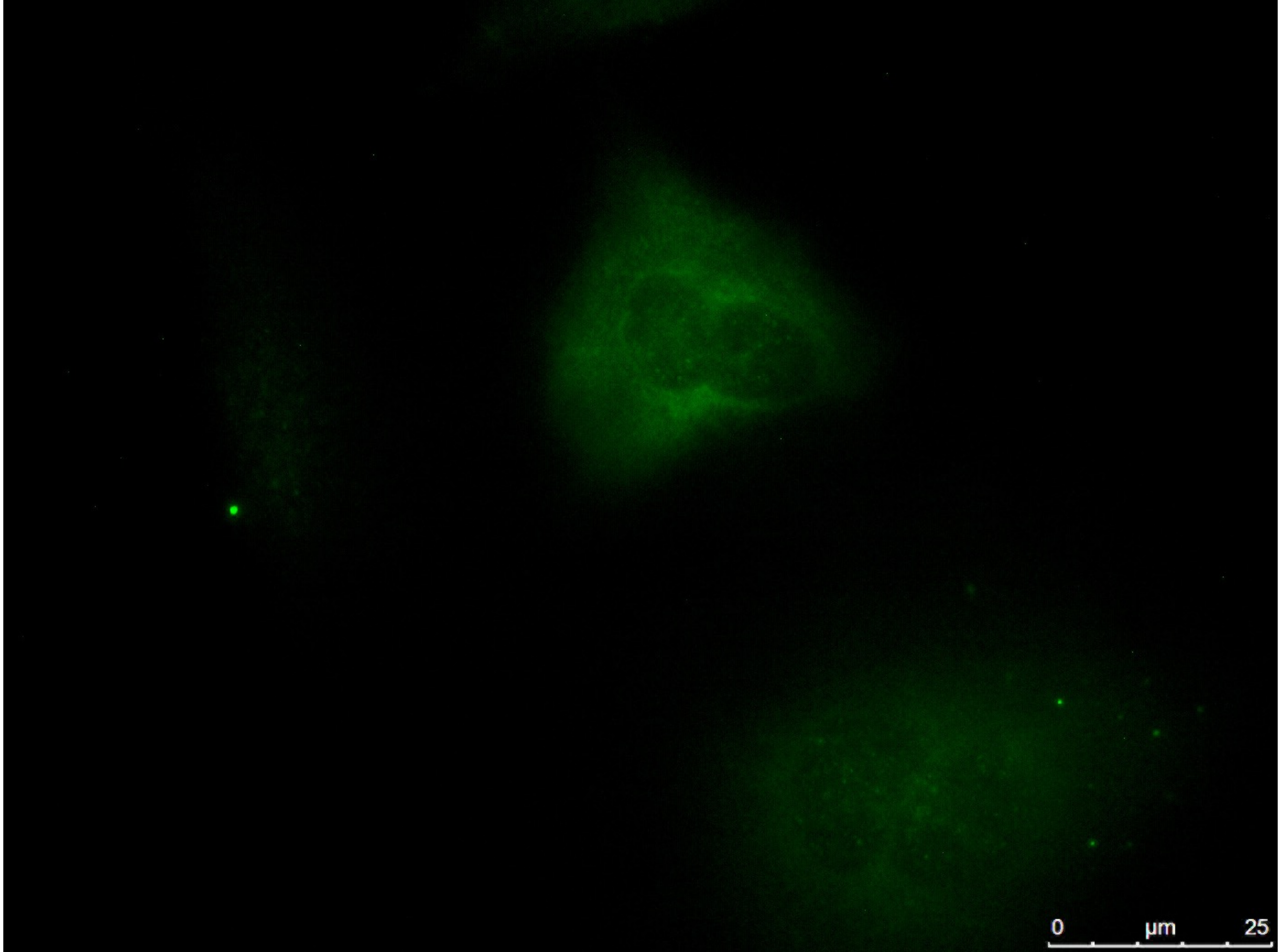

Fig 5B:
BI2536 mCherry

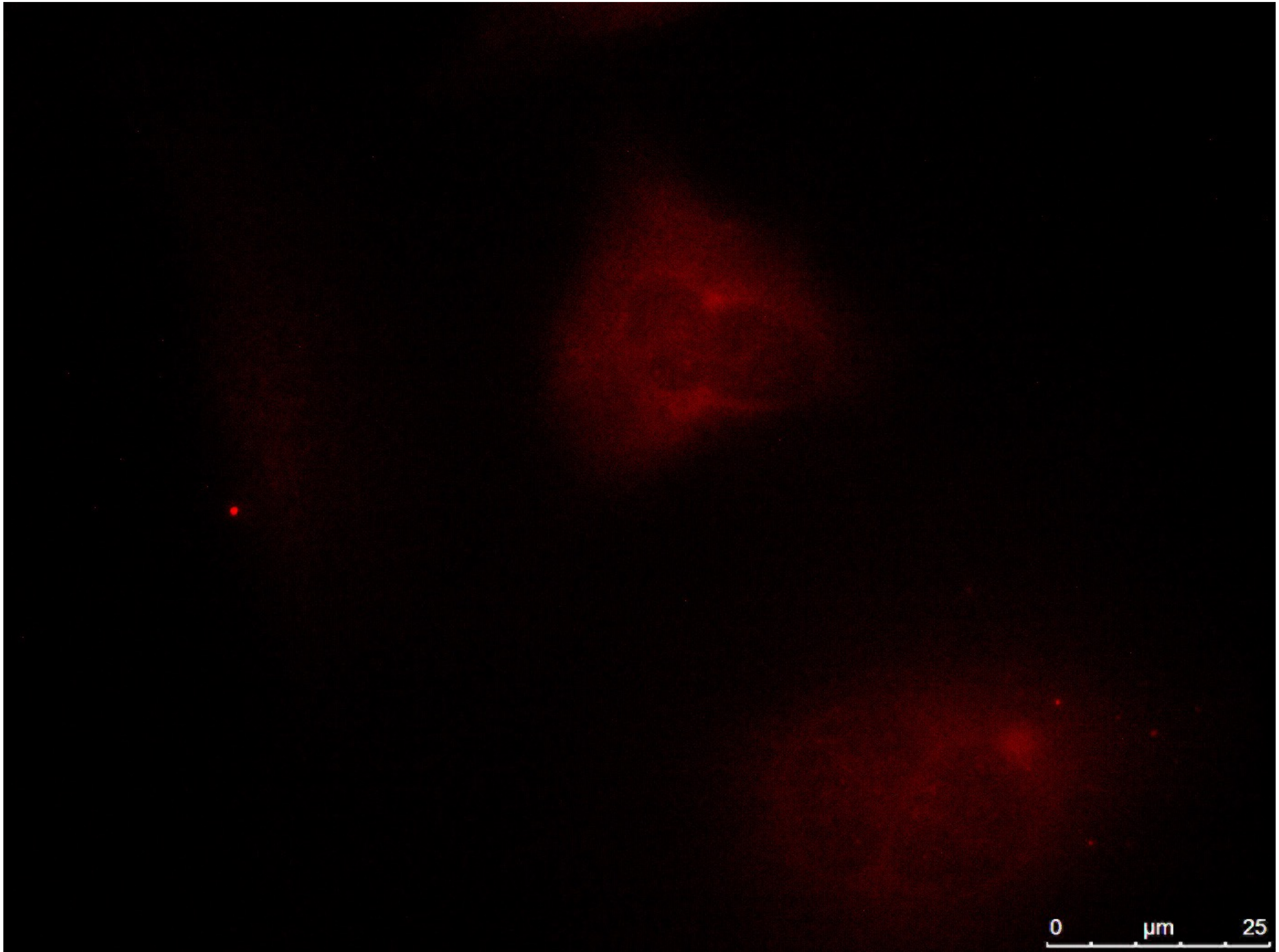

Fig 5B:
BI2536 Merge

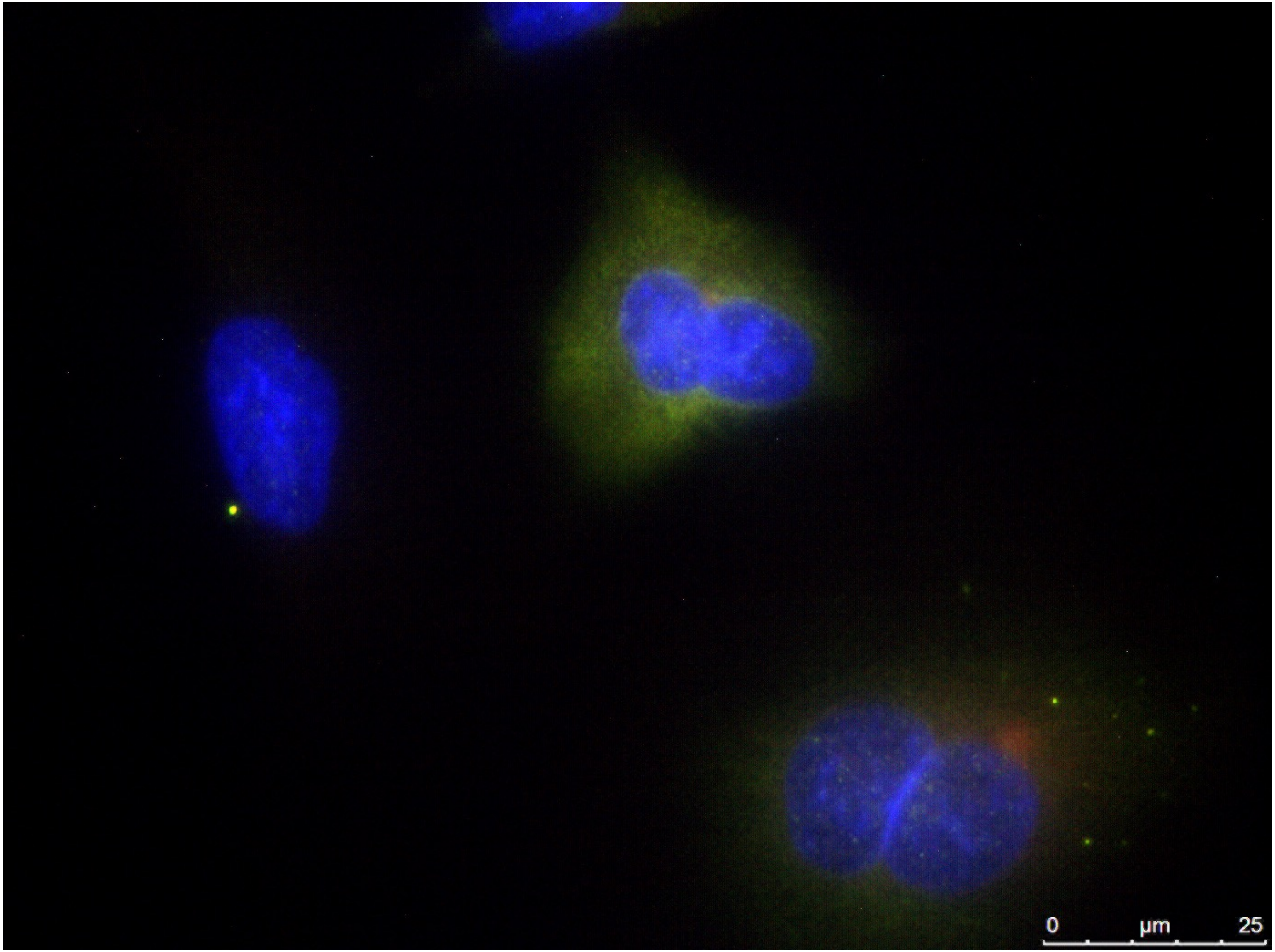

Fig 5B:
AZD+BI DAPI

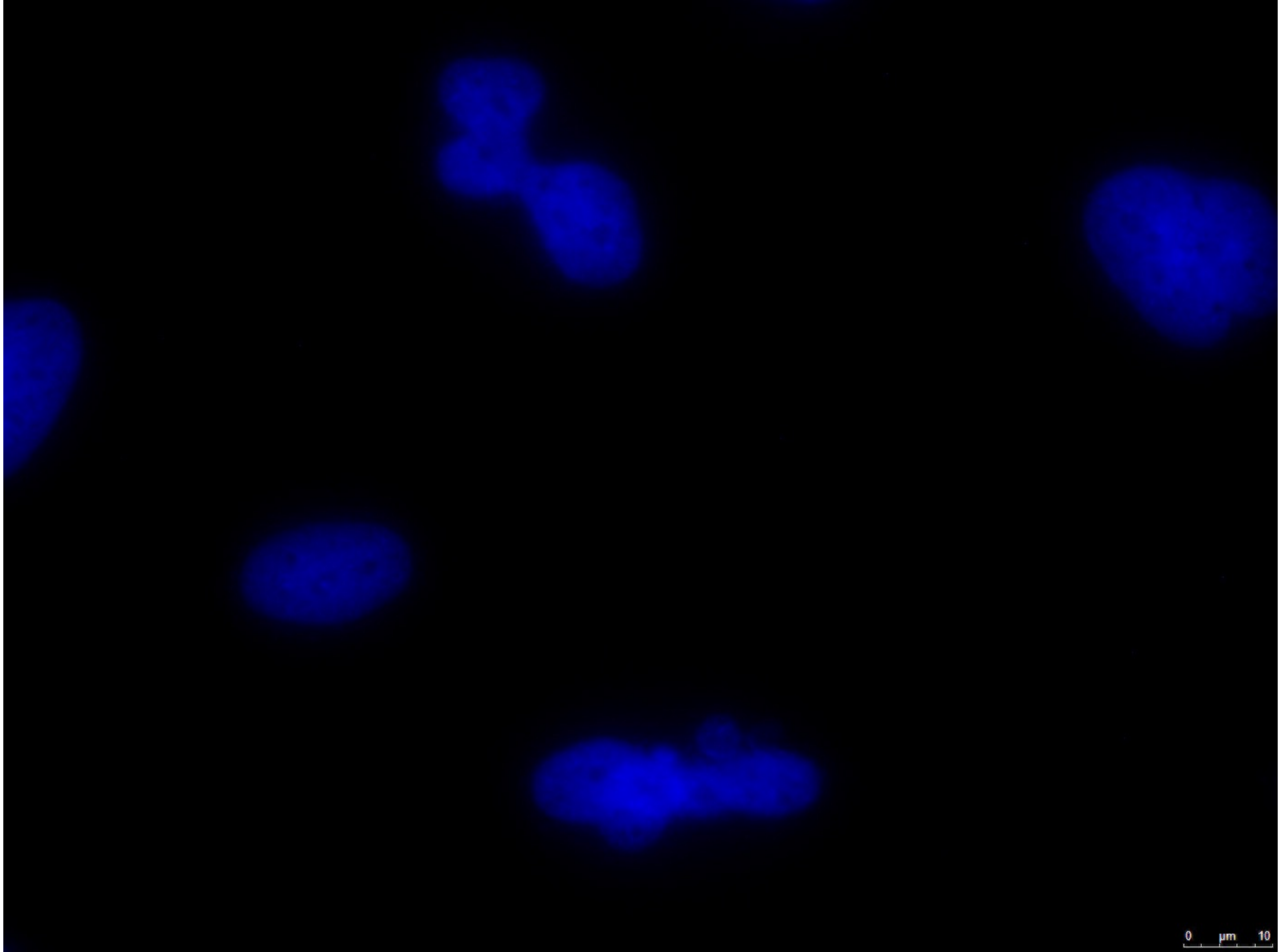

Fig 5B:
AZD+BI GFP

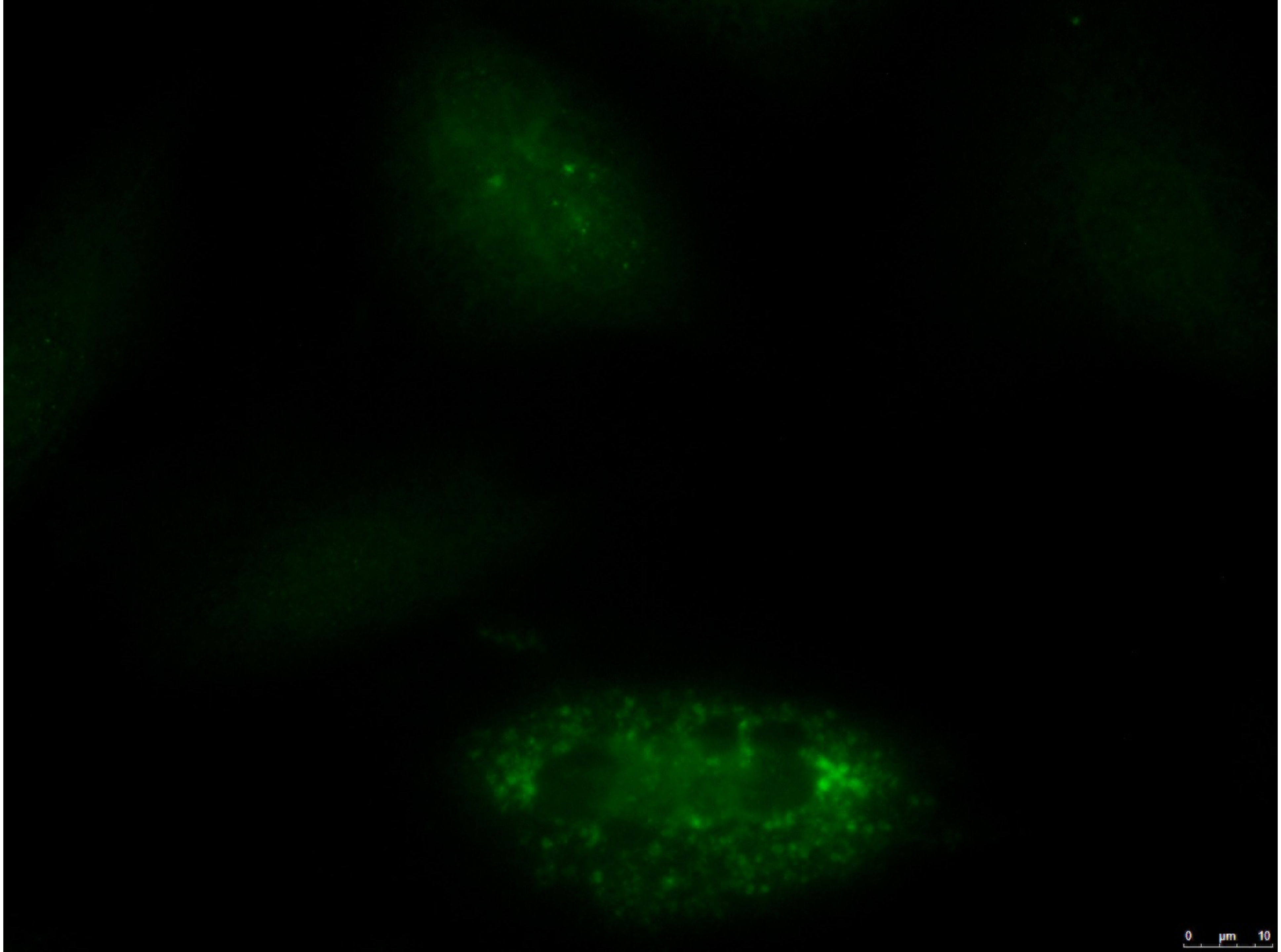

Fig 5B:
AZD+BI mCherry

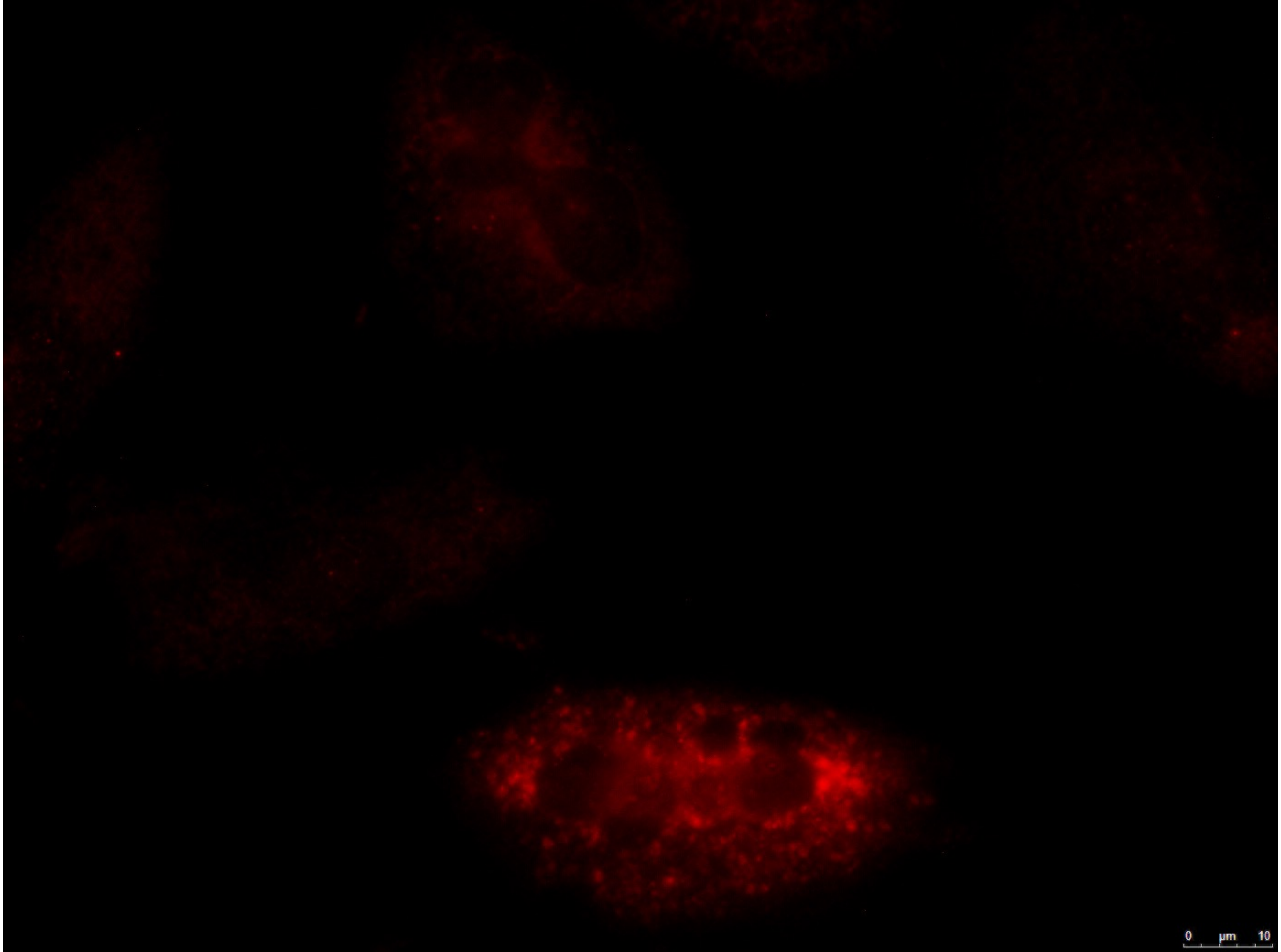

Fig 5B:
AZD+BI Merge

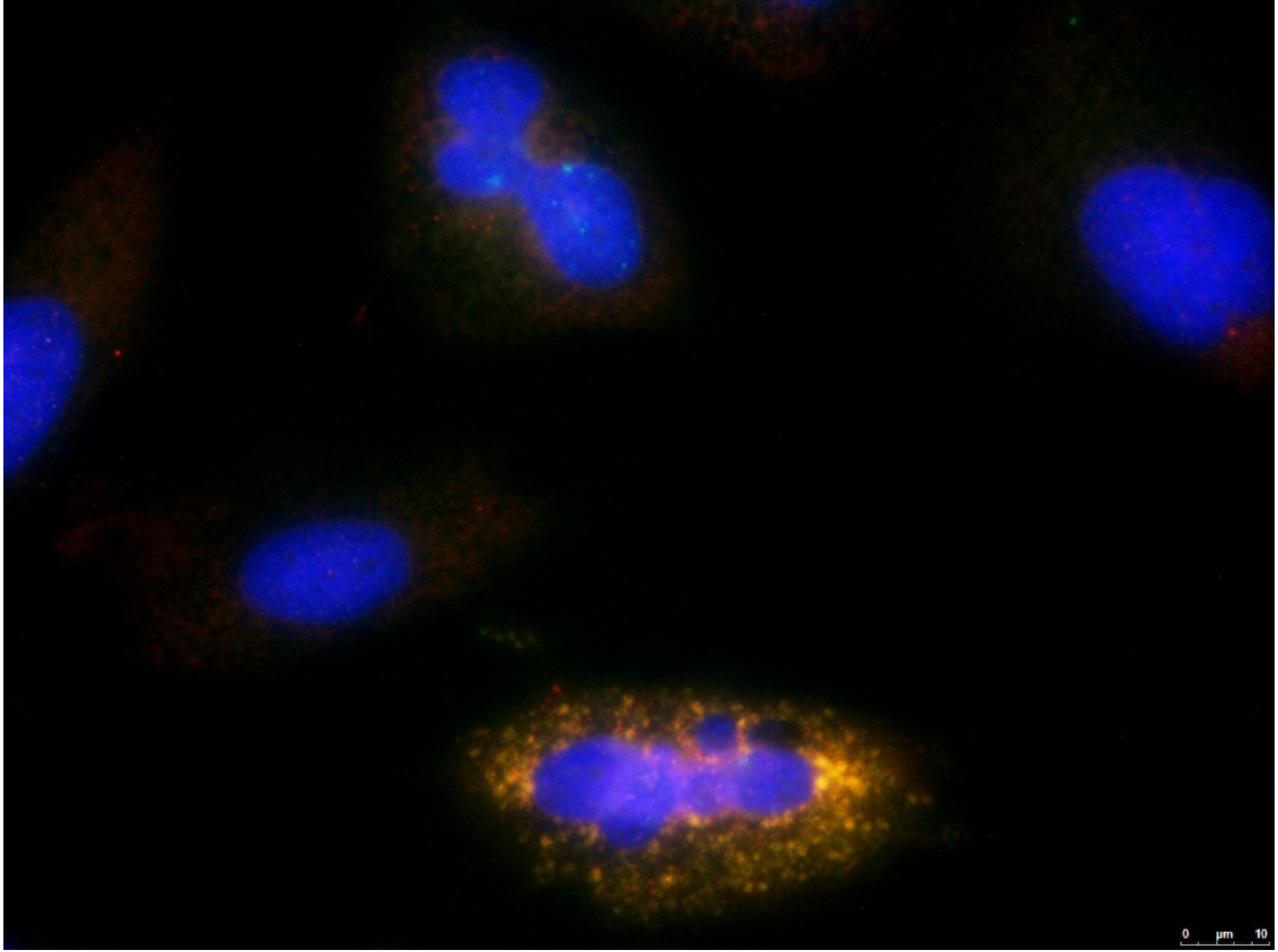

Supplement: Supplementary file 6 — Source Data for Figure 5 [file EMMM-13-e13193-s009.zip › Source data Figure 5B.pdf]
