# Supplementary material for: Synergistic effects of FGFR1 and PLK1 inhibitors target a metabolic liability in KRAS‐mutant cancer
Source: EMBO Mol Med. 2021 Aug 8;13(9):e13193. doi: 10.15252/emmm.202013193 (PMC8422071; doi:10.15252/emmm.202013193)

Fig S4

Fig. S4B

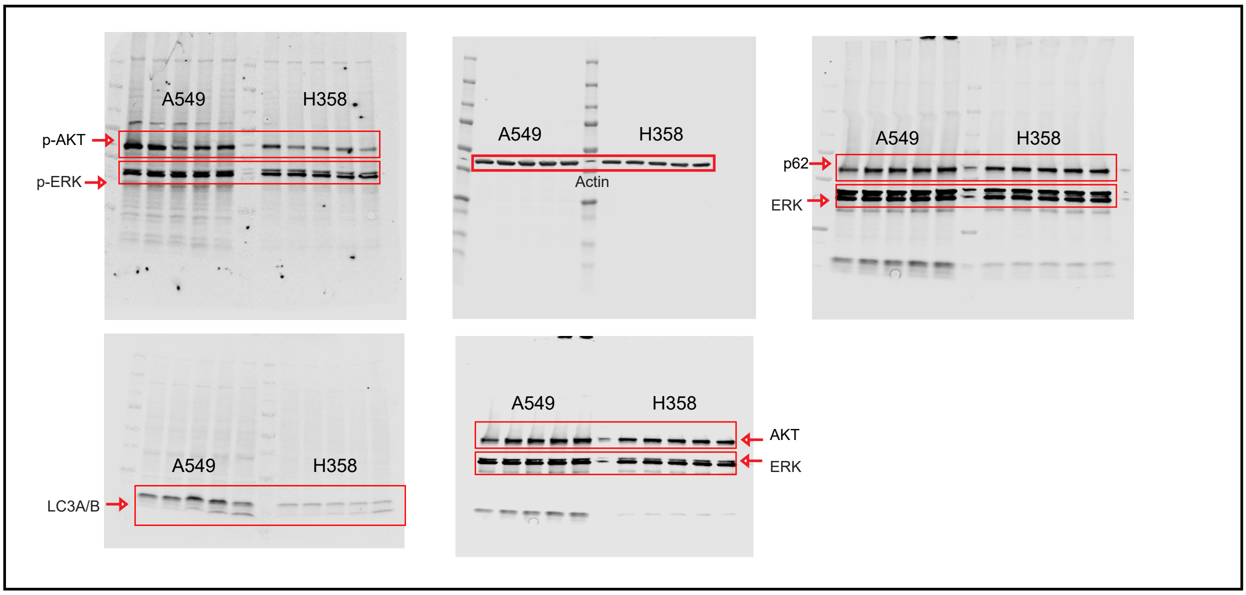

Fig. S4C

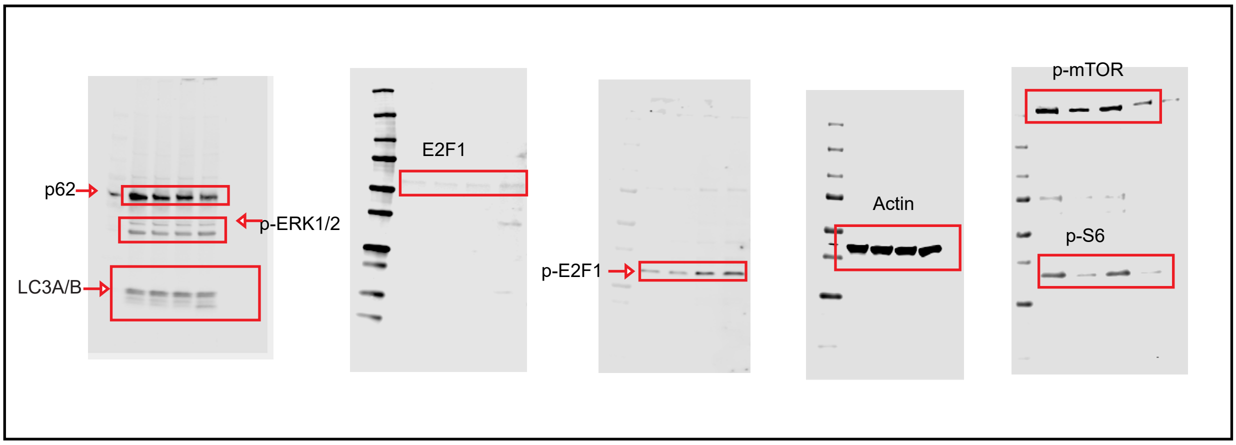

Fig. S4G

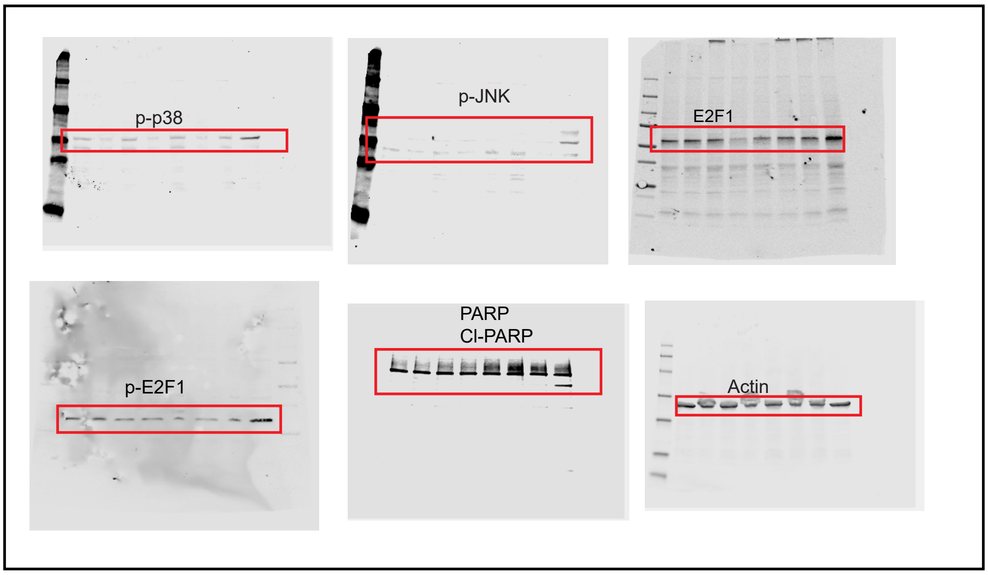

Supplement: Supplementary file 9 — Source Data for Appendix [file EMMM-13-e13193-s002.zip › Source Data Appendix Figure S4.zip!/source data_Appendix Fig. S4-1.pdf]
